# Doping effect of Ho on the structural, optical, ac electrical transport and magnetic properties of Mg–Zn ferrites nanoparticles

**DOI:** 10.1039/d6ra07670j

**Published:** 2026-10-16

**Authors:** Muhammad Samir Ullah, Kaniz Fatema Laizo

**Affiliations:** a Department of Physics, Bangladesh University of Engineering and Technology Bangladesh samirullah@phy.buet.ac.bd

## Abstract

Mg–Zn ferrites nanoparticles having the formula Mg_0.4_Zn_0.6_Ho_*x*_Fe_2−*x*_O_4_ (*x* = 0.00, 0.02, 0.04, 0.06 and 0.08) were synthesized using a hydrothermal method to investigate the effect of Ho substitution on their structural, ac electrical transport, optical and magnetic properties in this study. X-ray diffraction (XRD) analysis confirmed the formation of a cubic spinel structure for *x* ≤ 0.06, while a weak Ho_2_O_3_ secondary phase was detected at *x* = 0.08, suggesting the solubility limit of Ho ions within the spinel lattice. The experimental lattice parameters increased from 8.421 to 8.441 Å up to *x* = 0.06, while the crystallite size decreased with increasing Ho content. Transmission electron microscopy (TEM) analysis revealed that the average particle size decreases from 28.38 ± 0.47 to 20.66 ± 0.39 nm for *x* ≤ 0.06. The FTIR spectra indicated the characteristic metal–oxygen vibrational bands associated with the tetrahedral and octahedral sites. The tetrahedral band remained nearly unchanged at 532 cm^−1^, while the octahedral band shifted toward lower wavenumbers as the Ho content increases from *x* = 0.00 to *x* = 0.06, suggesting the preferential occupancy of Ho ions at the octahedral sites. Optical analysis demonstrated a reduction in the optical band gap energy from 1.62 to 1.47 eV for *x* ≤ 0.06, indicating enhanced visible-light absorption. An enhancement of coercivity (*H*_c_) is clearly observed and it increses from 33.9 (for *x* = 0.00) to 134.4 Oe (for *x* = 0.06). On the other hand, the magnetocrystalline anisotropy constant (*K*_a_) rapidly increased and it shows a higher value ∼3032 erg cm^−3^ for *x* = 0.06, while the value of *K*_a_ is 897 erg cm^−3^ for undoped sample. The significant increase of *K*_a_ along with *H*_c_ (for *x* = 0.06) indicates the enhancement of magnetic stability, which is promising applications in permanent magnets, magnetic recording media and high-density data storage devices. Frequency dependent dielectric constant (*ε*′) measurement has been displayed that an enhancement of *ε*′ is clearly evidenced on Ho doping and it is found to larger at *x* = 0.06. The inclusion of Ho in Mg–Zn ferrites shows lower value of dielectric losses, which could make them suitable in high-frequency operations. It is also seen that AC electrical conductivity reaches its maximum value for *x* = 0.06, which may be attributed to the enhancement of densification. Electrical modulus analysis shows a shift of the relaxation peak toward higher frequencies, suggesting a reduction in relaxation time. It is inferred that Mg_0.4_Zn_0.6_Ho_0.06_Fe_1.94_O_4_ exhibits the most optimized magnetic performance, characterized by the larger coercivity and enhanced magnetic anisotropy. In addition, its reduced optical band gap and favorable electrical response further extend its potential toward multifunctional optoelectronic and electromagnetic applications.

## Abstract 2

Mg–Zn ferrites nanoparticles having the formula Mg_0.4_Zn_0.6_Ho_*x*_Fe_2−*x*_O_4_ (*x* = 0.00, 0.02, 0.04, 0.06 and 0.08) were synthesized using a hydrothermal method.

## Graphical abstract

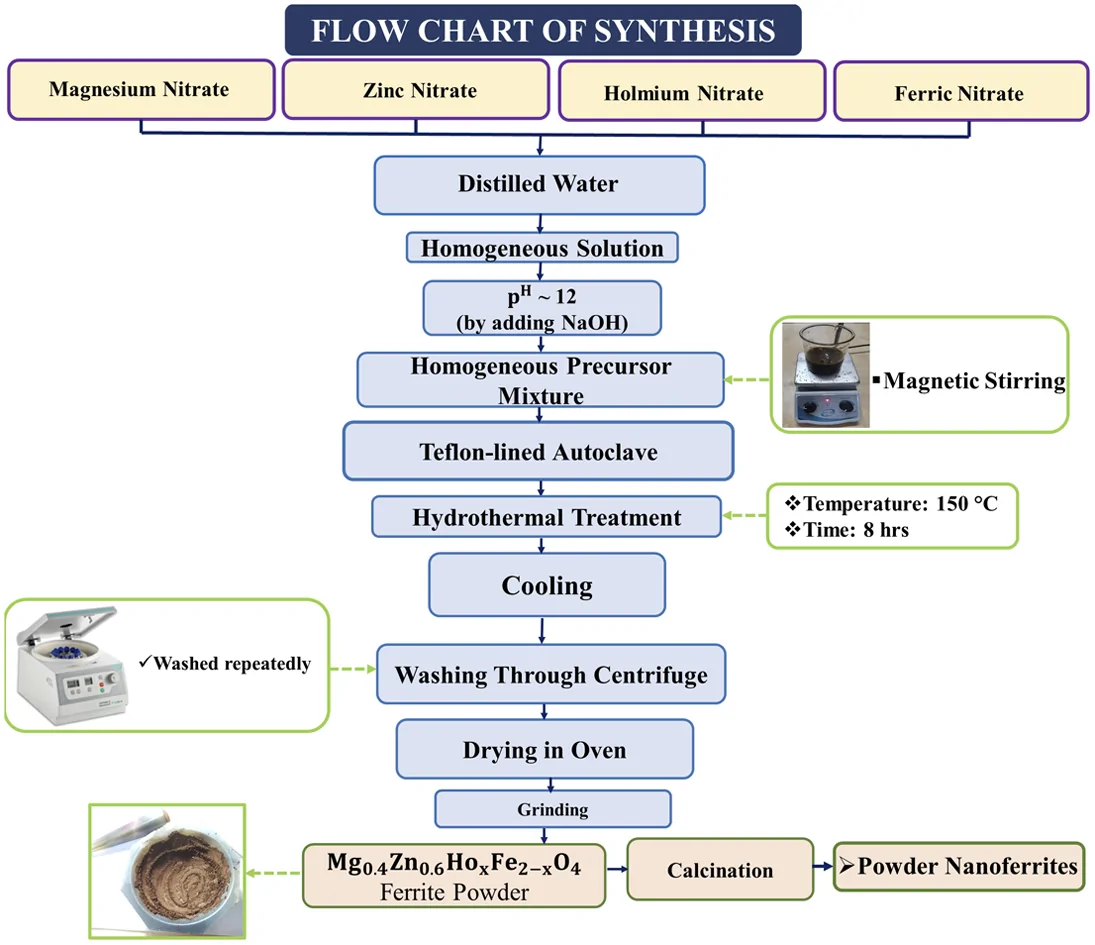

## 1 Introduction

In recent years, magnetic nanoparticles have gained considerable attention due to their unique size-dependent and tunable properties.^1^ Among various magnetic nanoparticles, spinel ferrites with the chemical formula MFe_2_O_4_ (where M represents a divalent metal ion such as Mg, Zn, Co, Ni, Mn *etc.*) are widely recognized due to their remarkable chemical stability, high mechanical hardness, moderate saturation magnetization and low eddy current losses.^2^ Nanocrystalline spinel ferrite possesses a large surface-to-volume ratio, which enhances the surface resistivity and catalytic efficiency while enabling the tuning of optical, magnetic and dielectric properties.^3,4^ These characteristics make them suitable for applications in high-frequency devices, telecommunication systems, sensors, microwave technologies and biomedical fields.^5–8^ Mg–Zn ferrite is an important mixed spinel ferrite which is utilized in a variety of electromagnetic devices, including high-density recording media, inductors, optoelectronic and photocatalytic applications.^9–12^ In Mg–Zn ferrite, Zn^2+^ ions have a strong preference at the tetrahedral (A) sites, pushing Fe^3+^ ions to the octahedral (B) sites, thereby strengthening the A–B superexchange interaction and enhancing the saturation magnetization. On the other hand, Mg^2+^ ions occupy at B-sites which contribute to improve the structural stability and electrical resistivity.^13^ Though higher saturation magnetization, Mg–Zn ferrite carry a reduced coercivity, weak magnetocrystalline anisotropy due to their soft nature and moderate dielectric performance which limits their use in advanced functional and energy storage systems. Rodriguez *et al.*^14^ observed the structural and magnetic properties of Mg_1−*x*_Zn_*x*_Fe_2_O_4_ (*x* = 0.0–0.9). They synthesized the samples through a sol–gel method. They found that the saturation magnetization (*M*_s_) increases with increment of Zn substitution and reaches a higher value. However, despite this improved magnetization, the coercivity and remanent magnetization (*M*_r_) remain very low. This low value of coercivity indicate poor magnetic hardness that causes magnetic instability, higher energy loss which become a burden in applications such as magnetic storage, electromagnetic shielding and energy storage systems. Singh *et al.*^15^ successfully synthesized Mg_*x*_Zn_1−*x*_Fe_2_O_4_ (*x* = 0.5, 0.6, 0.7) with superparamagnetic behavior *via* using co-precipitation technique. Yet, they observed that the coercivity values ranges from 18.67 to 23.52 Oe, which is very low and it limits their potentiality in hard magnetic applications. To address these issues, rare-earth ions can be introduced into the spinel lattice. Doping of rare-earth ions in spinel ferrite play significant role to modify the optical, dielectric and magnetic properties. Rare-earth ion doping in spinel ferrite has been extensively studied to enhance the multifunctional properties of spinel ferrites.^16,17^ Previous studies have also employed Raman spectroscopy to investigate the vibrational characteristics of ferrite nanocrystals and to obtain information related to their local structural environment and metal–oxygen bonding. Such studies demonstrate the usefulness of Raman spectroscopy as a complementary technique for understanding structural features of spinel ferrites. As observed previously from the Raman analysis, the variations in the Raman-active modes support the modification of the local structural environment and provide complementary evidence of local structural distortion associated with Ho incorporation at the B-site.^18^ The incorporation of rare-earth may improve magnetocrystalline anisotropy by inducing lattice distortion and hindering domain wall motion which increases both coercivity and remanence. Taleb *et al.*^19^ studied the magnetic response of Ho-doped Ni–Cu spinel ferrites *via* a sol–gel autocombustion technique. They revealed that Ho-doping in Ni–Cu ferrites increases coercivity to a higher value due to enlargement of the hysteresis loop area, resulting in a shift toward hard magnetic behavior. Furthermore, Pessoni *et al.*^20^ investigated Ho-substituted CoFe_2−*x*_Ho_*x*_O_4_ ferrites prepared using the forced hydrolysis technique. They reported that Ho incorporation effectively tuned the optical properties of ferrite system. Specifically, the substitution of Ho increased the reduced mass of the metal–oxygen bonds, leading to a decrease in the optical band gap energy. To the best knowledge no study has been reported on Ho-doped Mg–Zn ferrites nanoparticles. The present work aims to conduct a detailed investigation into the effect of Ho substitution on Mg–Zn ferrite nanoparticles, where Ho induced structural modification and expected to enhance magnetic anisotropy and coercivity while simultaneously improving the optical and dielectric property.

## 2 Experimental procedure

### 2.1 Synthesis of Mg_0.4_Zn_0.6_Ho_*x*_Fe_2−*x*_O_4_ nanoparticles

Nanocrystalline Mg_0.4_Zn_0.6_Ho_*x*_Fe_2−*x*_O_4_ (*x* = 0.00, 0.02, 0.04, 0.06 and 0.08) spinel ferrites were synthesized *via* a hydrothermal technique, as schematically illustrated in Fig. 1. The hydrothermal reaction was performed in a temperature-controlled oven equipped with an internal thermocouple, providing temperature stability within ±1 °C throughout the synthesis process. Stoichiometric quantities of magnesium nitrate hexahydrate [Mg(NO_3_)_2_·6H_2_O], zinc nitrate hexahydrate [Zn(NO_3_)_2_·6H_2_O], ferric nitrate nonahydrate [Fe(NO_3_)_3_·9H_2_O] and holmium nitrate pentahydrate [Ho(NO_3_)_3_·5H_2_O] were subsequently mixed under continuous magnetic stirring to obtain a homogeneous solution. A sodium hydroxide (NaOH) solution was then introduced dropwise until the p^H^ reached ∼13. The resulting suspension was transferred into a Teflon-lined stainless-steel autoclave, sealed and maintained 150 °C for 8 h in the oven. Upon completion of the reaction, the autoclave was allowed to cool naturally to room temperature. The obtained precipitate was collected and repeatedly washed with deionized water and ethanol. The purified product was dried at 80 °C overnight. Then the dried sample was grinded to obtain a fine powder. Finally, the powder was calcined at 600 °C for 4 h to promote the formation of a well-crystallized single-phase spinel ferrite.

**Fig. 1:**
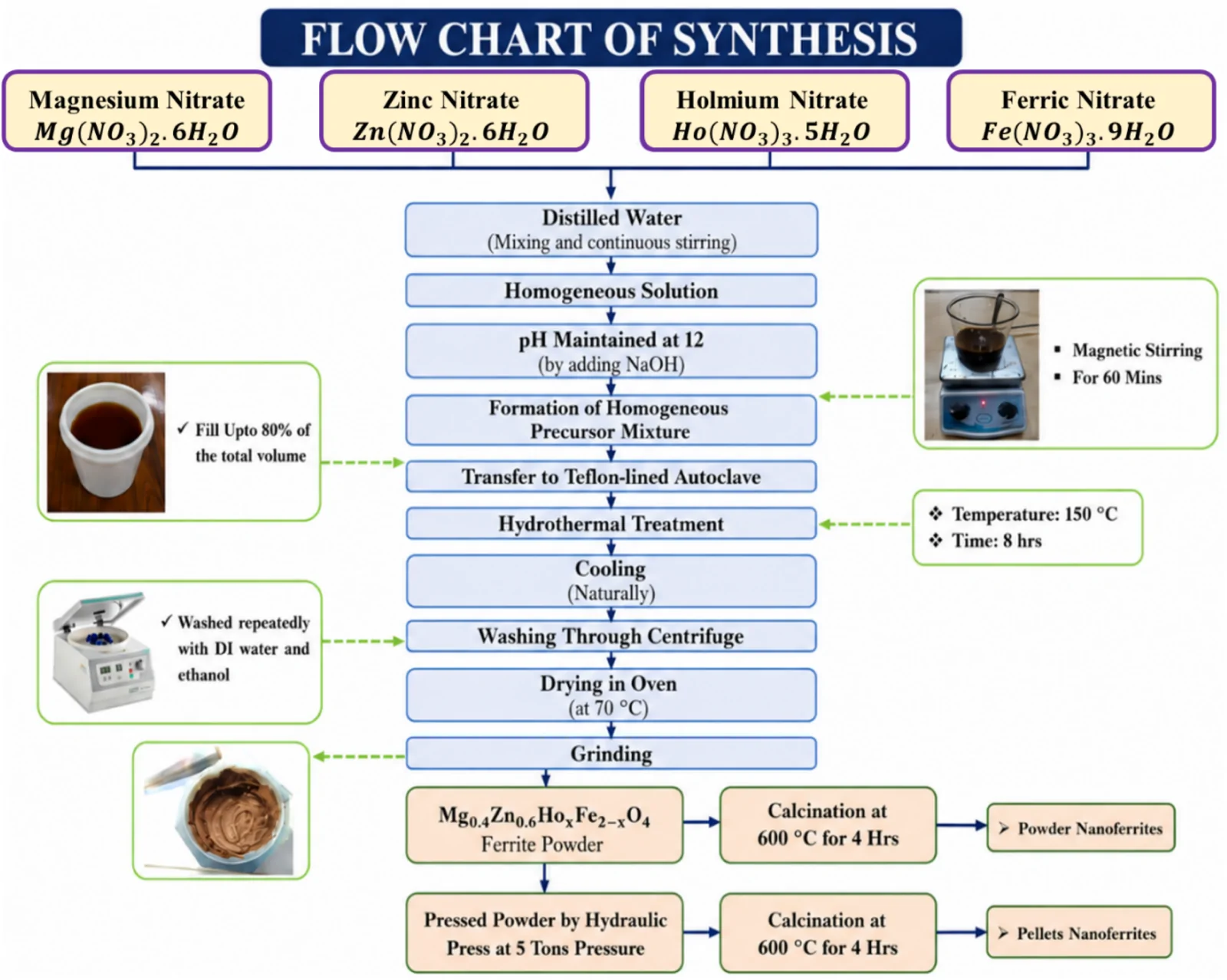
Flow chart of synthesis of the sample.

### 2.2 Characterization technique

The crystal structure and phase purity of the synthesized nanoparticles were investigated using a PANalytical Empyrean X-ray diffractometer with Cu-Kα radiation (*λ* = 1.5406 Å), operated at 45 kV and 40 mA. The diffraction patterns were recorded over a 2*θ* range of 20°–80° with a scanning rate of 2°/min. The surface morphology was examined using a JEOL JSM-7600 F field-emission scanning electron microscope (FESEM), while the particle size and microstructural feature were analyzed using a Talos F200 X transmission electron microscope (TEM). Fourier transform infrared (FTIR) spectra were recorded using a PerkinElmer spectrometer in the wavenumber range 350–900 cm^−1^. The magnetic properties were measured at room temperature using a DynaCool Physical Property Measurement System (PPMS) by recording the magnetic hysteresis (M − H) loops. Dielectric measurement were carried out using a Wayne Kerr 6500 B Precisio Impedance Analyzer at room temperature. The optical absorption spectra were obtained using a HITACHI U-2900 UV-vis double-beam spectrophotometer over the wavelength range 300–800 nm.

The structural parameters were determined from the X-ray diffraction (XRD) data using standard crystallographic relations. The interplanar spacing (*d*) was calculated according to Bragg's law expressed as:

2.1 *nλ* = 2d sin *θ*

The lattice parameter (*a*_exp_) was calculated using the following relation:2.2
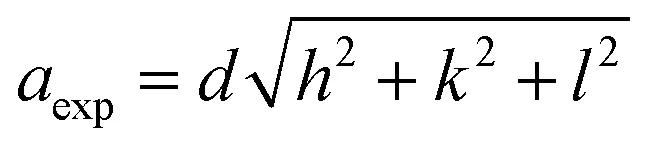
where (*hkl*) denotes the Miller indices of the corresponding crystallographic planes.

## 3 Results and discussion

### 3.1 Structural properties

#### 3.1.1 Crystal structure

The Rietveld refinement of X-ray diffraction (XRD) patterns for Mg_0.4_Zn_0.6_Ho_*x*_Fe_2−*x*_O_4_ (*x* = 0.00, 0.02, 0.04, 0.06, and 0.08) is displayed in Fig. 2. For each composition, the observed (*Y*_obs_), calculated (*Y*_cal_) and difference (*Y*_obs_ − *Y*_cal_) profiles are plotted along with the allowed Bragg reflection positions. The close overlap between the observed and calculated patterns, together with the flat, low-amplitude difference curves, confirms that all the diffraction peaks indexed to the (220), (311), (400), (422), (511) and (440) planes have been satisfactorily fitted to the single-phase cubic spinel structure with space group *Fd*-3*m*.^21^ The impurity peak has not been detected for *x* ≤ 0.06. For higher Ho content (*x* = 0.08), an additional low-intensity reflection marked with an asterisk (*) near the (220) peak has been identified as a secondary Ho_2_O_3_ phase, indicating that the solubility limit of Ho ion in the spinel lattice.^22^ The Rietvelt phase analysis indicates that the *x* = 0.08 sample consists of approximately 97% spinel phase and 3% Ho_2_O_3_ secondary phase (as shown in Fig. 2 inset). The refined structural parameters such as lattice parameter (*a*_exp_), unit cell volume (*V*), reliability factors (*R*_p_, *R*_wp_), and goodness-of-fit (*χ*^2^) are summarized in Table 1. The value of *a*_exp_ increases from 8.421 (for *x* = 0.00) to 8.441 Å (for *x* = 0.06), which can be attributed to the larger ionic radius of Ho ion (≈0.901 Å) compared to Fe ion (≈0.645 Å). This variation confirms that Ho ions have been successfully incorporated into the spinel structure at lower doping levels.^23^ A corresponding increase in unit cell volume (*V*) from 597.01 to 601.50 Å^3^ over the same range. At *x* = 0.08, however, both *a* and *V* decrease slightly, which occurs due to the onset of Ho_2_O_3_ phase.^24^

**Fig. 2:**
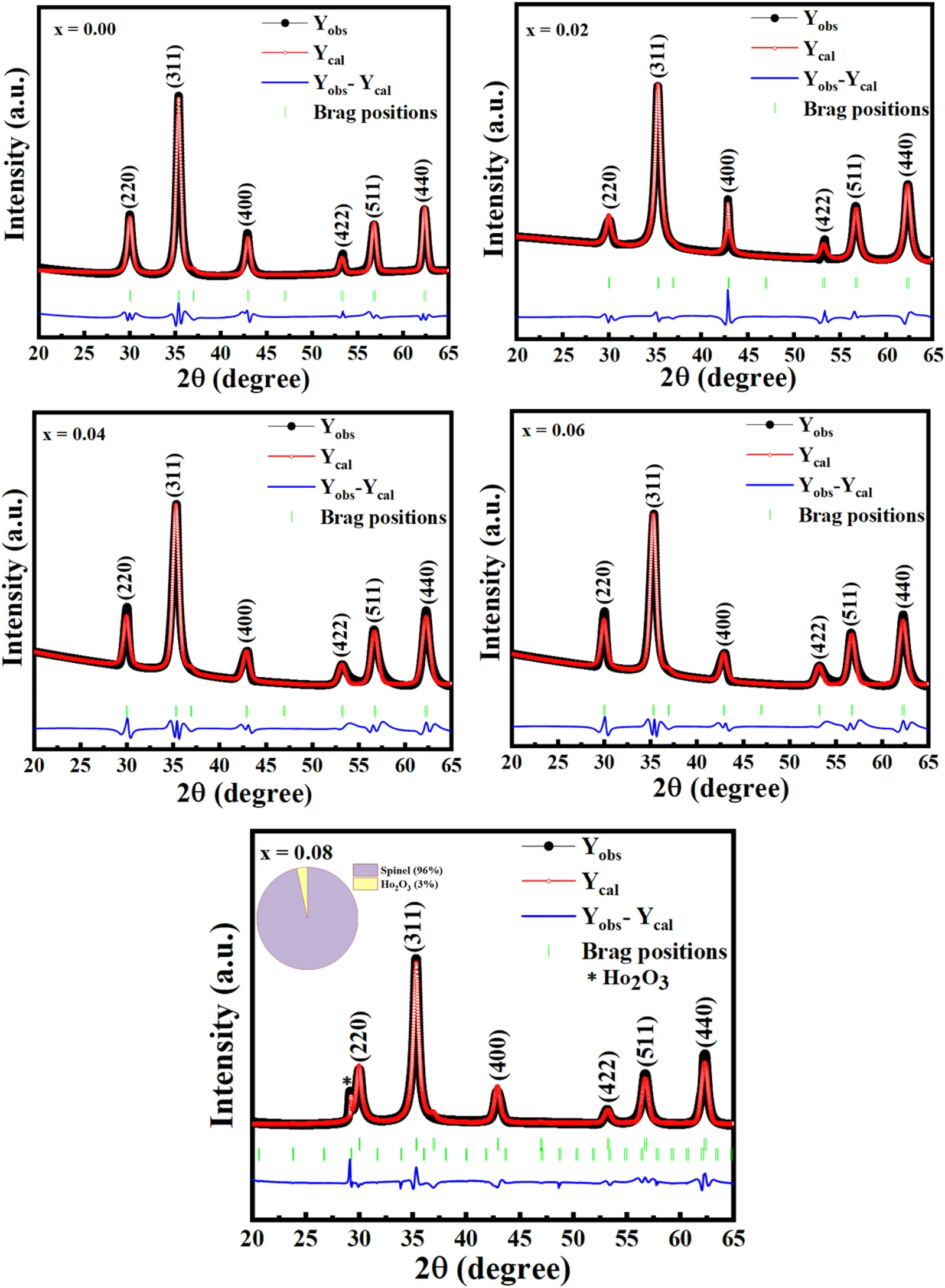
Rietveld refinement of XRD patterns for different Mg_0.4_Zn_0.6_Ho_*x*_Fe_2−*x*_O_4_.

**Table 1:** Lattice parameter (*a*_exp_), unit cell volume, (*V*), *R*-factors (*R*_p_, *R*_wp_) and goodness of fit (*χ*^2^) and space group from Rietveld analysis

Composition (*x*)	*a*_exp_ (Å)	*V* (Å^3^)	*R*_p_ %	*R*_wp_ %	*χ* ^2^ %	Space group
0.00	8.421	597.01	18.6	11.1	1.06	*Fd*-3*m*
0.02	8.429	599.05	15.3	13.1	1.22	*Fd*-3*m*
0.04	8.436	600.38	18.4	13.3	1.09	*Fd*-3*m*
0.06	8.441	601.50	19.7	15.2	1.36	*Fd*-3*m*
0.08	8.430	598.87	18.5	14.7	1.18	*Fd*-3*m*

The hopping lengths of the tetrahedral (A-site) and octahedral (B-site) denoted as *L*_A_ and *L*_B_, respectively, has been calculated and the estimated values are given in Table 2. It indicates that the cation hopping length increase with increasing Ho content up to *x* = 0.06. This can be explained through the replacement of smaller Fe ions by larger Ho ions which causes lattice expansion leading to increment the separation between the neighboring cations. However, for *x* = 0.08, a reduction in both of the hopping length is observed which can be attributed by the solubility limit of Ho ions in ferrite lattice, which results formation an additional secondary phase.

**Table 2:** Experimental lattice parameter (*a*_exp_), theoretical lattice parameter (*a*_th_), ionic radius of tetrahedral site (*r*_A_), ionic radius of octahedral site (*r*_B_), hopping length of A-site (*L*_A_), hopping length of B-site (*L*_B_), oxygen positional parameter (*u*) and interplanar spacing (*d*_XRD_ and *d*_TEM_)

Composition (x)	*a*_exp_ (Å)	*a*_th_ (Å)	*r*_A_ (Å)	*r*_B_ (Å)	*L*_A_ (Å)	*L*_B_ (Å)	*u* (Å)	*d*_XRD_ (nm)	*d*_TEM_ (nm)
0.00	8.421	8.425	0.712	0.666	3.648	2.978	0.387	0.2541	0.2547
0.02	8.429	8.430	0.712	0.668	3.650	2.981	0.387	0.2543	0.2548
0.04	8.436	8.436	0.712	0.670	3.653	2.982	0.387	0.2546	0.2551
0.06	8.441	8.442	0.712	0.672	3.655	2.984	0.387	0.2589	0.2558
0.08	8.430	8.449	0.712	0.675	3.650	2.980	0.386	0.2540	0.2545

Table 3 shows the atomic positions, Wyckoff sites and site occupancies obtained from the refinement, describing the cation distribution across the spinel sub-lattices. Zn^2+^ and a fraction of Fe^3+^ ions occupy at the tetrahedral (8a) site, while Mg^2+^, the remaining Fe^3+^, and the doped Ho^3+^ ions occupy in the octahedral (16d) site; oxygen anions occupy the 32e position. As the Ho content increases from *x* = 0.02 to 0.08, its occupancy at the 16d site rises proportionally, accompanied by a corresponding decrease in the Fe occupancy at the same site, confirming that Ho^3+^ substitutes for Fe^3+^ preferentially at the octahedral position. This cation redistribution at the B-site (16d) influence the super exchange interactions (Fe^3+^–O^2−^–Fe^3+^) and consequently, the magnetic behavior of the samples. The VSM results are in well agreement with this cation redistribution through the Néel two-sublattice model.

**Table 3:** Atomic positions, Wyckoff sites, and site occupancies for the Ho-doped Mg–Zn ferrite

Sample	Atom	Wyckoff	*x*	*y*	*z*	Occupancy
*x* = 0.00	Zn	8a	0.125	0.125	0.125	0.60
	Fe	8a	0.125	0.125	0.125	0.40
	Mg	16d	0.500	0.500	0.500	0.40
	Fe	16d	0.500	0.500	0.500	1.60
	O	32e	0.387	0.387	0.387	1.00
*x* = 0.02	Zn	8a	0.125	0.125	0.125	0.60
	Fe	8a	0.125	0.125	0.125	0.40
	Mg	16d	0.500	0.500	0.500	0.40
	Fe	16d	0.500	0.500	0.500	1.58
	Ho	16d	0.500	0.500	0.500	0.02
	O	32e	0.387	0.387	0.387	1.00
*x* = 0.04	Zn	8a	0.125	0.125	0.125	0.60
	Fe	8a	0.125	0.125	0.125	0.40
	Mg	16d	0.500	0.500	0.500	0.40
	Fe	16d	0.500	0.500	0.500	1.56
	Ho	16d	0.500	0.500	0.500	0.04
	O	32e	0.387	0.387	0.387	1.00
*x* = 0.06	Zn	8a	0.125	0.125	0.125	0.60
	Fe	8a	0.125	0.125	0.125	0.40
	Mg	16d	0.500	0.500	0.500	0.40
	Fe	16d	0.500	0.500	0.500	1.54
	Ho	16d	0.500	0.500	0.500	0.06
	O	32e	0.387	0.387	0.387	1.00
*x* = 0.08	Zn	8a	0.125	0.125	0.125	0.60
	Fe	8a	0.125	0.125	0.125	0.40
	Mg	16d	0.500	0.500	0.500	0.40
	Fe	16d	0.500	0.500	0.500	1.52
	Ho	16d	0.500	0.500	0.500	0.08
	O	32e	0.386	0.386	0.386	1.00

#### 3.1.2 Cation distribution

The possible cation distribution of the synthesized samples can be represented as:(Zn_0.6_Fe_0.4_)[Mg_0.4_Ho_*x*_Fe_1.6−*x*_]where the parentheses ( ) and square [ ] brackets denote tetrahedral-A and octahedral-B sites, respectively. The distribution is based on the well-established site preferences of the constituent cations in spinel ferrites, where Zn^2+^ ions preferentially occupy at the A-sites, while Mg^2+^ and Ho^3+^ ions predominantly reside in the B-sites. Fe^3+^ ions are distributed between both crystallographic sites to maintain charge neutrality and structural stability. The average ionic radii of tetrahedral (*r*_A_) and octahedral (*r*_B_) sites have been calculated by using the following equations:

3.1 *r*_A_ = 0.6*r*_Zn^2+^_ + 0.4*r*_Fe^3+^_

3.2
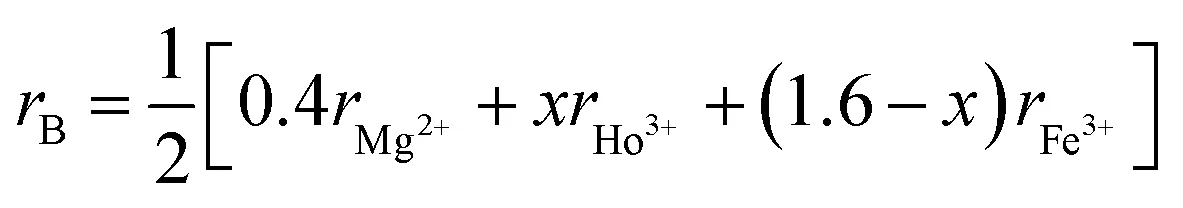


The calculated values are presented in Table 2. It is observed that the tetrahedral radius (*r*_A_) remains nearly constant at ∼0.712 Å across all compositions, whereas the octahedral radius (*r*_B_) gradually increases from 0.666 Å to 0.675 Å with higher Ho substitution. This increase arises from the replacement of Fe^3+^ ions (*r* = 0.645 Å) by the larger Ho^3+^ ions (*r* = 0.901 Å) at the octahedral sites. The enlarged B-site radius induces local lattice distortion and elongates the metal–oxygen bond length. The systematic rise in *r*_B_ confirms the successful incorporation of Ho^3+^ into the spinel lattice. Furthermore, the expansion of the B-site radius alters interionic distances and bond geometry, thereby influencing structural parameters and superexchange interaction, which ultimately affect the magnetic and electrical properties of the ferrite system. To further assess the effect of cation substitution, the theoretical lattice parameter (*a*_th_) has been calculated according to the relation:^25^3.3
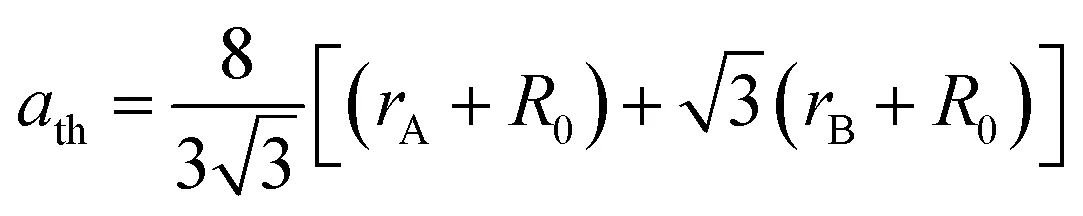
where *R*_0_ represents the radius of the oxygen ion (*R*_0_ = 1.32 Å). The estimated values are represented in Table 2. The gradual enhancement of *r*_B_ contributes directly to the increment in the theoretical lattice parameter with an increase in Ho concentration. The theoretical lattice parameter (*a*_th_) shows close agreement with the experimental lattice parameter (*a*_exp_), supporting the proposed cation distribution and confirming the successful incorporation of Ho ions into the spinel lattice. The consistency between theoretical and experimental parameters indicates that the synthesized ferrites are largely preserve the spinel crystal framework despite the introduction of spinel lattice. However, at higher Ho concentration, a noticeable deviation between (*a*_th_) and (*a*_exp_) is observed, which can be explained by the limited solubility of the Ho ions in spinel lattice. The oxygen positional parameter (*u*), which is calculated using following expression:3.4
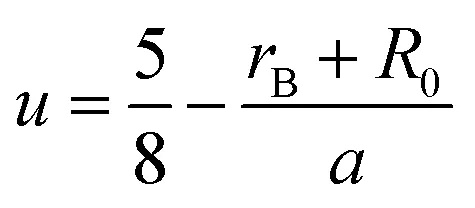
where *R*_0_ represents the ionic radius of oxygen. The oxygen positional parameter (*u*) provides information about the degree of distortion in the spinel lattice. For an ideal cubic spinel, *u* = 0.375, which corresponding to an undistorted oxygen framework with ideal cation-oxygen coordination. Any deviation from this ideal value indicates structural distortion. It is observed from Table 2 that the calculated oxygen positional parameter of all synthesized samples (*u* ≈ 0.389) is very close to the ideal value, suggesting that the cubic spinel structure is well preserved despite the incorporation of the dopant.

#### 3.1.3 Bond lengths and angles

To observe the deeper insight of the influence of Ho substitution in Mg–Zn spinel ferrites, various bond-length, edge-length, interionic distance parameter and bond angles have been analyzed. The bond lengths at tetrahedral and octahedral sites have been calculated from the following relations;3.5
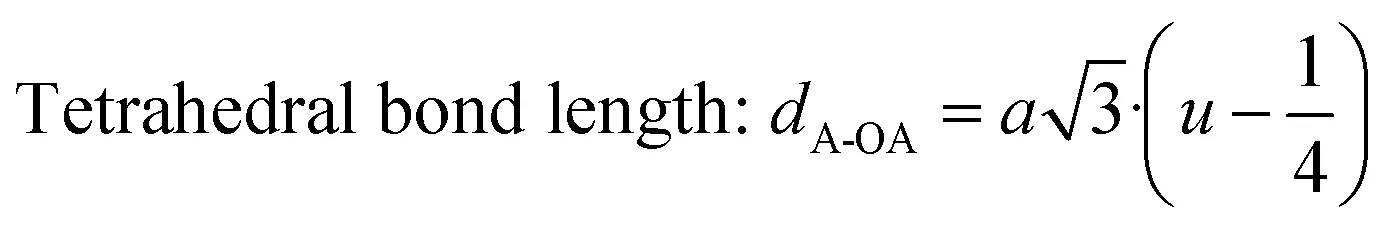
3.6
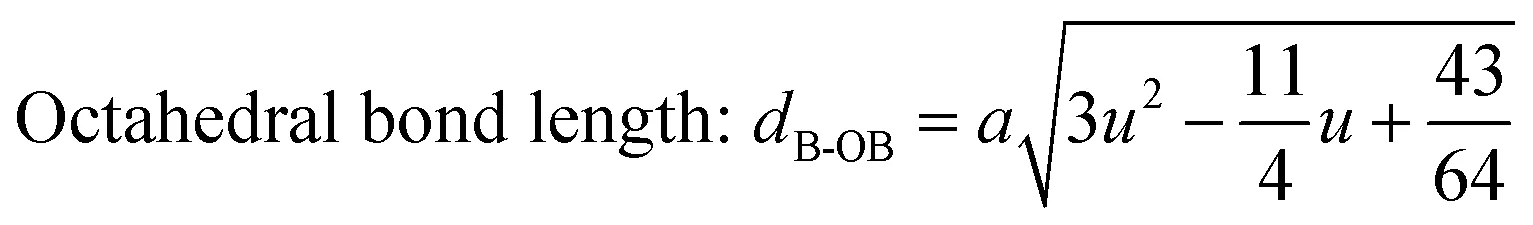


The tetrahedral edge length (*d*_AE_), the shared octahedral edge length (*d*_BE_), and the unshared length (*d*_BEU_) have been calculated by using equation:3.7
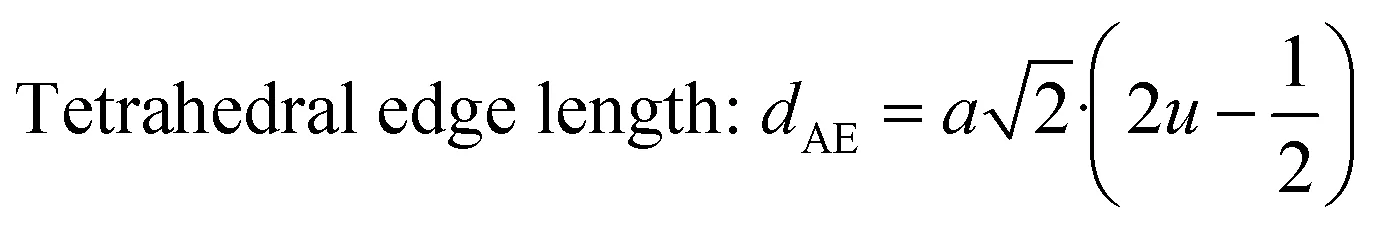
3.8
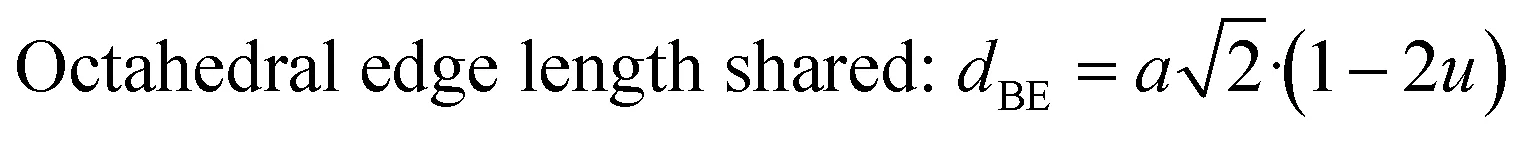
3.9
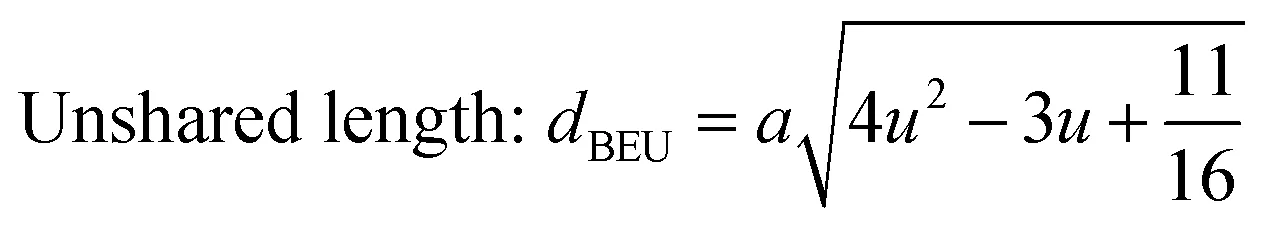


The calculated values have been summarized in Table 4. It is observed that all these parameters gradually increase with Ho content up to *x* = 0.06 and subsequently decrease at *x* = 0.08. The initial increase can be attributed to the substitution of larger Ho ions for smaller Fe ions at the octahedral sites. This substitution at octahedral sites expands the surrounding oxygen coordination environment and enlarges the tetrahedral and octahedral oxygen polyhedral, resulting in progressive lattice expansion and enhanced local distortion up to *x* = 0.06. The increment in *d*_A-OA_, *d*_B-OB_, *d*_AE_, *d*_BE_, and *d*_BEU_ increased the interionic distance between magnetic cations and oxygen ions, which reduces the overlap between Fe (3d) and O (2p) orbitals, thereby weakening the A–O–B superexchange interactions. This reduced magnetic exchange coupling between the A and B sublattice leads to a lower saturation magnetization (*M*_s_).^26^ The interionic separations have been evaluated for both cation–anion pairs [metal ions – oxygen ions: (*p*, *q*, *r*, *s*)] and cation–cation pairs [metal ions – metal ions: (*b*, *c*, *d*, *e*, *f*)]. The corresponding distances have been determined and the values are presented in Table 4. The calculated interionic distance parameters exhibit a gradual increase with increasing Ho concentration up to *x* = 0.06, indicating lattice expansion and the increased cation–cation separation weakens Fe^3+^–O^2−^–Fe^3+^ superexchange interaction. However, at *x* = 0.08, a slight decrease in most interionic distances is observed, which may be attributed to structural rearrangement and partial strain relaxation caused by the emergence of a secondary phase.^27^3.10
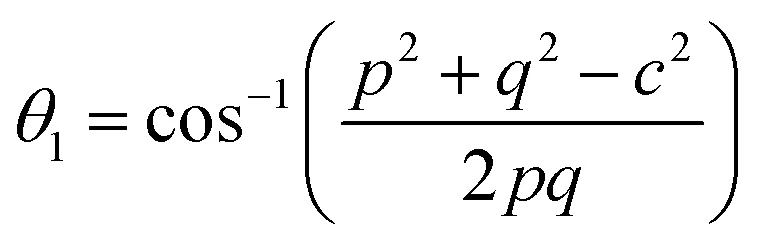
3.11
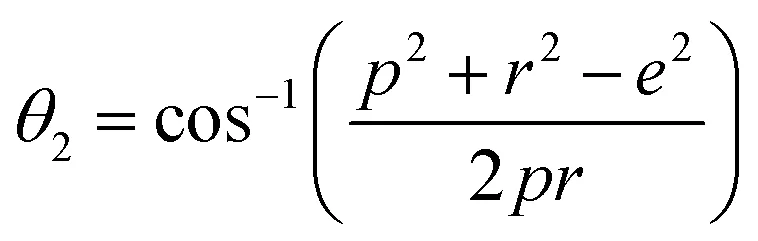
3.12
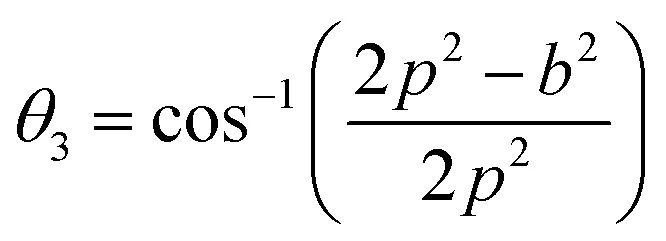
3.13
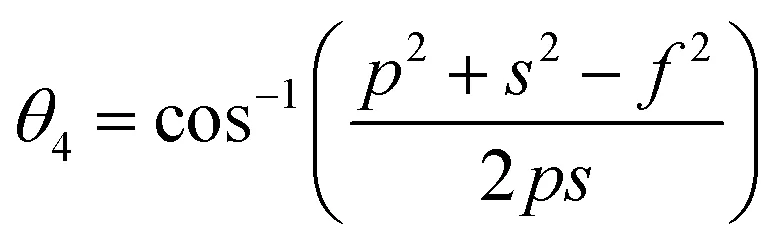
3.14
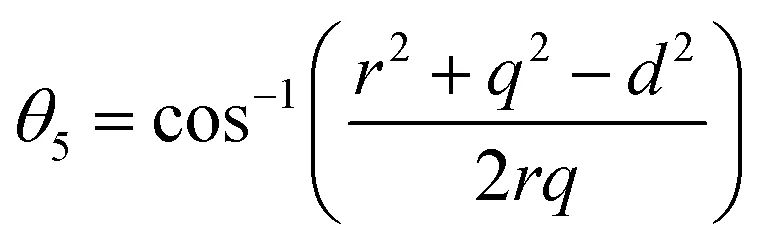


**Table 4:** Different structural parameters *i.e.* bond angles and bond lengths

Ho ions conc.	0.00	0.02	0.04	0.06	0.08
*d*_A-OA_	2.034	2.037	2.040	2.042	2.039
*d*_B-OB_	1.990	1.992	1.993	1.994	1.991
*d*_AXE_	3.322	3.327	3.331	3.334	3.330
*d*_BXE_	2.631	2.633	2.634	2.635	2.630
*d*_BEU_	2.987	2.990	2.992	2.994	2.991
*b*	2.977	2.980	2.982	2.985	2.980
*c*	3.491	3.494	3.497	3.499	3.495
*d*	3.646	3.652	3.653	3.655	3.650
*e*	5.469	5.475	5.479	5.482	5.475
*f*	5.156	5.162	5.166	5.169	5.162
*p*	1.983	1.984	1.985	1.986	1.983
*q*	2.034	2.037	2.040	2.042	2.039
*r*	3.886	3.901	3.906	3.910	3.905
*s*	3.716	3.721	3.724	3.726	3.721
*θ*_1_	120.660	120.642	120.626	120.607	120.627
*θ*_2_	134.357	134.290	134.234	134.167	134.298
*θ*_3_	97.292	97.326	97.353	97.387	97.344
*θ*_4_	126.865	126.871	126.877	126.884	126.876
*θ*_5_	67.671	67.626	68.589	68.545	68.601

Furthermore, the bond angles corresponding to these interionic distances (*θ*_1_, *θ*_2_, *θ*_3_, *θ*_4_, *θ*_5_) are calculated using the prescribed relations and the results are listed in Table 4. The calculated bond angles reveal that *θ*_1_, *θ*_2_ and *θ*_5_ decrease gradually whereas *θ*_3_ and *θ*_4_ increase with increasing Ho content up to *x* = 0.06. These variation in the bond angles indicate that Ho substitution introduces lattice distortion and modifies the local cation-oxygen-cation bond geometry within the spinel structure. The decrease in *θ*_1_ and *θ*_2_ suggests that the A–O–B linkage becomes more distorted, while the increase in *θ*_3_ and *θ*_4_ indicates a slight rearrangement of the B–O–B linkage. Similarly, the reduction in *θ*_5_ reflects the distortion of the A–O–A bond geometry. Such structural modifications influence the magnetic superexchange interactions. Since *θ*_1_ and *θ*_2_ are associated with the dominant A–B superexchange pathway, their decrease weakens the orbital overlap between A-site and B-site magnetic ions through the intervening oxygen ion, leading to a weaker A–B exchange interaction. On the other hand, the increase in *θ*_3_ and *θ*_4_ may slightly strengthen the B–B interaction. Consequently, the weakening of the dominant A–B superexchange interaction is expected to reduce the saturation magnetization (*M*_s_).^28^ However, for *x* = 0.08 sample, the bond angles show a different trend than the lower Ho content. This deviation can be attributed to the formation of a secondary Ho_2_O_3_ phase after exceeding the solubility limit of Ho in the spinel lattice. As a result, a portion of the Ho ions segregates from the spinel structure, partially relieving the lattice distortion and altering the local cation arrangement, which consequently modifies the bond angles and the corresponding magnetic exchange interaction.

#### 3.1.4 Crystallite size

The crystallite size represents the average size of a single coherently diffracting crystal domain. The crystallite size of Mg_0.4_Zn_0.6_Ho_*x*_Fe_2−*x*_O_4_ (*x* = 0.00, 0.02, 0.04, 0.06, 0.08) has been calculated by using Debye Scherrer equation:^29^3.15
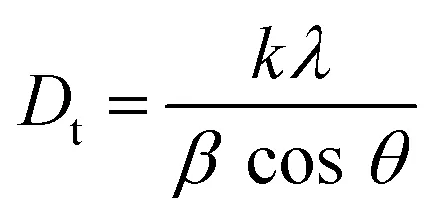
where *k* represents the shape factor (0.9), *λ* is the wavelength of Cu-K_α_ radiation (1.5406 Å), *β* denotes the full width at half maximum (FWHM) of the prominent (311) diffraction peak, and *θ* corresponds to the Bragg diffraction angle. The calculated crystallite sizes are listed in Table 5. It is observed that the crystallite size decreases gradually from 15.47 (for pristine ferrite) to 9.79 nm (for *x* = 0.06). For more accuracy modified Scherrer method (MSM), Williamson–Hall (W–H) and Size–Strain Plot (SSP) methods have also been used to calculate the crystallite size of the synthesized samples and the results has been enlisted in Table 5. The modified Scherrer method utilizes linear fitting of multiple diffraction peak, thereby minimizing errors associated with individual peak broadening.^30^ The corresponding equation is:3.16
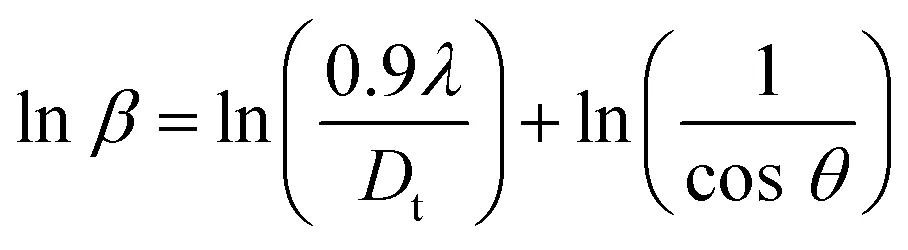


**Table 5:** Crystallite size (*D*_t_) and micro-strain (*∈*) estimated using various analytical methods

Composition (*x*)	Scherrer method	Modified Scherrer method	Williamson–Hall method		Size strain method	
	*D*_t_ (nm)	*D*_t_ (nm)	*D*_t_ (nm)	*ε* (%)	*D*_t_ (nm)	*ε* (%)
0.00	15.47	18.12	23.36	0.115	28.32	0.471
0.02	13.22	15.27	20.92	0.126	24.13	0.495
0.04	12.07	14.33	18.24	0.313	23.04	0.523
0.06	9.79	12.03	15.51	0.384	21.12	0.534
0.08	13.98	14.77	23.11	0.231	21.53	0.509

The corresponding linear fitting plots of ln *β versus*
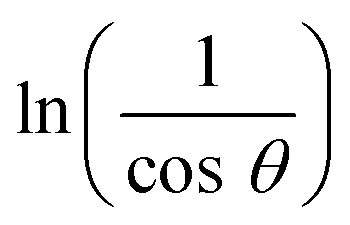
 is shown in Fig. 3(a). The intercepts of the Modified Scherrer plots represent the term 
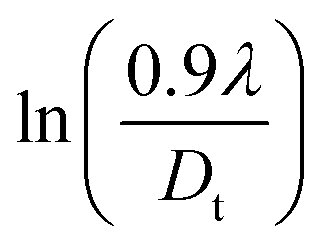
, from which the crystallite size is determined directly. Consequently, slightly larger crystallite sizes than the Scherrer method is found and the crystallite size decrease from 18.12 (*x* = 0.00) to 12.03 nm (*x* = 0.06). Unlike these two methods, the Williamson–Hall method considers both crystallite size and lattice strain assuming isotropic peak broadening through the relation:^31^3.17
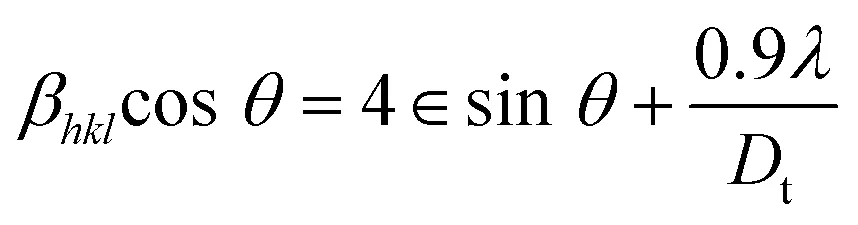
Here, *β*_*hkl*_ represents the FWHM of the diffraction peak corresponding to (*hkl*) plan and *∈* denotes the lattice strain. The linear fitting plots represented in Fig. 3(b) enable the simultaneous determination of the lattice strain *∈* from the slope and crystallite size *D*_t_ from the *y*-intercept. From Table 5, it is seen that the crystallite size decreases from 23.36 to 15.51 nm and the lattice strain increases from 0.115% to 0.384% with the increase of Ho concentration up to *x* = 0.06. Furthermore, the Size–Strain Plot (SSP) method provides the most reliable estimation by separating the size and strain contributions and assigning greater statistical weight to diffraction peaks with lower uncertainty. The corresponding relation between the crystallite size and lattice strain according to this model is related by:^32^3.18
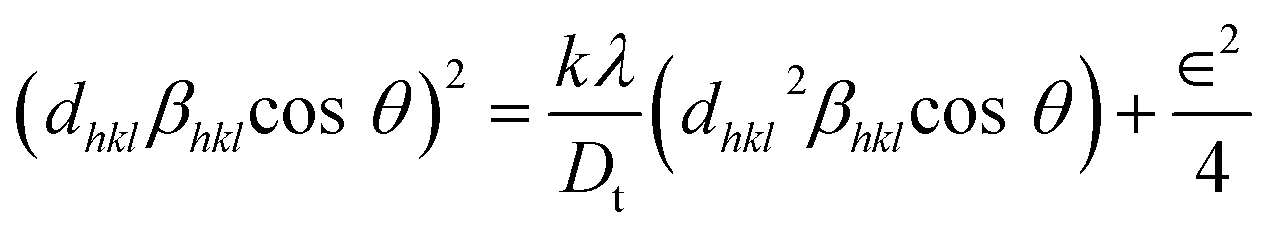
In this analysis, *d*_*hkl*_ corresponds to the lattice spacing of the respective (*hkl*) planes. The data are linearly fitted by plotting *d*_*hkl*_^2^*β*_*hkl*_cos *θ* against (*d*_*hkl*_*β*_*hkl*_cos *θ*)^2^, as depicts in Fig. 3(c). The lattice strain is extracted from the *y*-intercept of the fitted line, while the crystallite size is evaluated from its slope. From the estimated value obtained from this method represented in Table 5, it is seen that the crystallite size evaluated by this method is larger among all methods and the value of *D*_t_ decreases from 28.32 to 21.12 nm, whereas the lattice strain increases from 0.471% to 0.534% with Ho content up to *x* = 0.06. From all method it is confirm that the crystallite size decreases with Ho substitution up to *x* = 0.06. This reduction can be attributed to the substitution of larger Ho ions for Fe ions in spinel lattice introduces lattice distortion and enhances the internal microstrain (*∈*) as confirmed by Williamson–Hall and Size–Strain plot analyses. Moreover, the formation of Ho–O bonds requires higher bond formation energy than Fe–O bonds, which suppresses the crystallization process and hinders crystallite growth.^33^ The accumulated lattice strain and the reduced crystallization kinetics, decreases the coherence length of the diffracting domains, resulting smaller crystallites with increasing Ho content.^34^ However, a slight increase in crystallite size is observed for the *x* = 0.08 samples in all methods. This behavior can be explained by the appearance of a secondary Ho_2_O_3_ phase which indicates that the solubility limit of Ho in the spinel lattice has been exceeded which relieves the lattice distortion and results in slight increase in the crystallite size.^35^

**Fig. 3:**
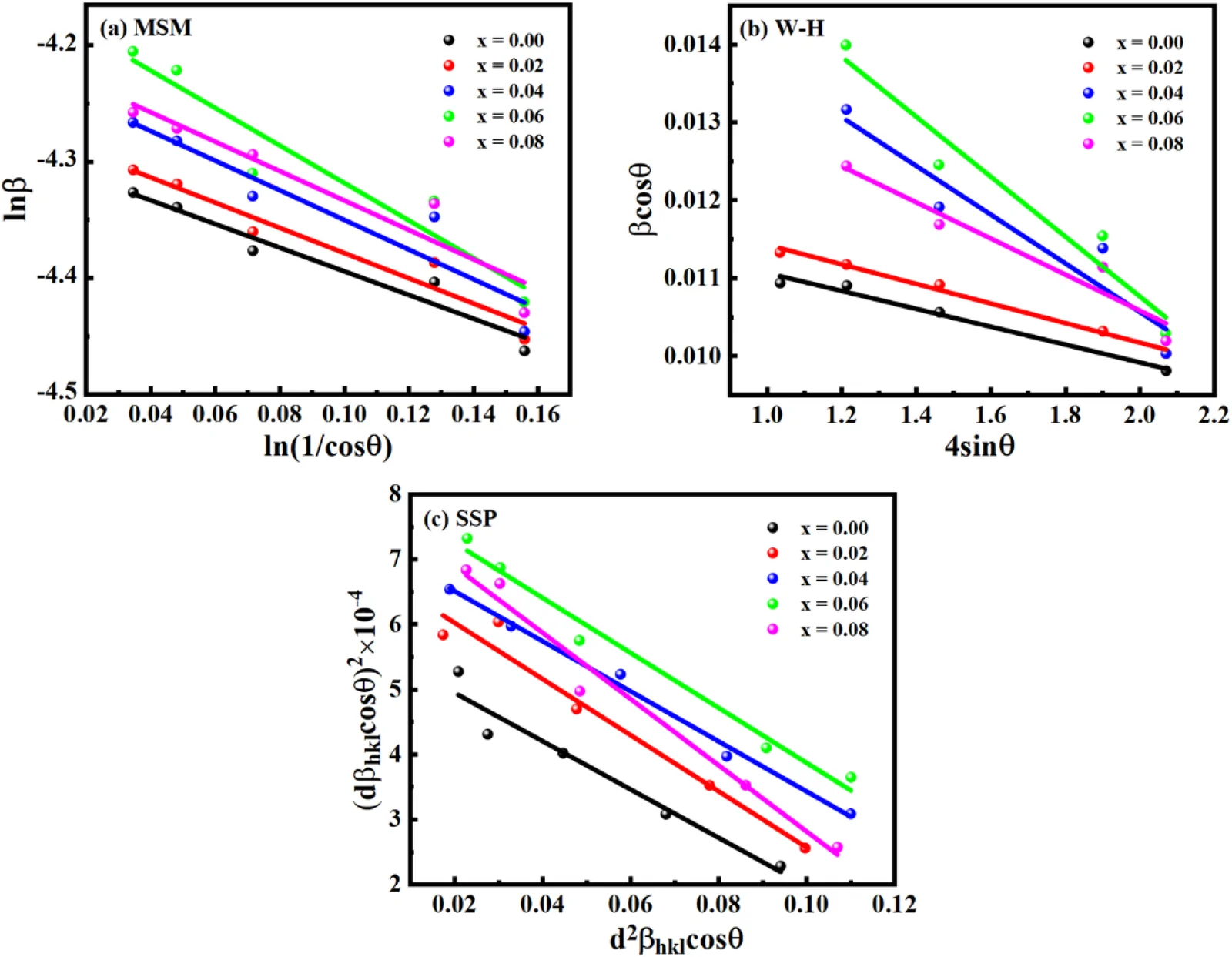
(a) Modified Scherer plot, (b) Williamson–Hall plot, and (c) size strain plot.

The X-ray density (*ρ*_*x*_), bulk density (*ρ*_B_) and porosity (*P* %) of the synthesized samples are calculated and presented in Table 6. The porosity decreases as Ho content increase from 0.00 to 0.06, while bulk density increases from 3.07 to 3.56 g cm^−3^ up to *x* = 0.06. The initial increase in bulk density may be attributed to improved densification of the ferrite matrix.^36^ However, at higher Ho concentration *x* = 0.08, limited solubility of Ho ions in the spinel lattice leads to a formation of a secondary phase which results an increase in porosity.^37^

**Table 6:** X-ray density (*ρ*_*x*_), bulk density (*ρ*_B_) and porosity (*P* %)

Composition (*x*)	*ρ*_*x*_ (g cm^3^)	*ρ*_B_ (g cm^3^)	*P* (%)
0.00	4.982	3.074	38.3
0.02	5.025	3.442	31.5
0.04	5.067	3.517	30.6
0.06	5.114	3.651	28.6
0.08	5.192	3.484	32.9

### 3.2 TEM analysis

The TEM micrographs are shown in Fig. 4. All compositions consists of nearly spherical nanoparticles with slight agglomeration, which is observed in magnetic ferrite nanoparticles due to their high surface energy and magnetic dipole–dipole interactions.^38^ The average particle size has been estimated and it is observed that the particle size calculated for the pristine sample is 28.38 ± 0.47 nm. It decreases with increasing Ho content to 20.66 ± 0.39 nm up to *x* = 0.06, indicating that Ho substitution effectively suppresses particle growth during synthesis process. This reduction in particle size may be attributed to the lattice distortion introduced by the larger ionic radius of Ho compared with Fe which hinders atomic diffusion. However, a slight increase in the average particle size was observed for the *x* = 0.08 sample. The observed variation in particle size with Ho doping agrees well with the variation in crystallite size obtained from XRD analysis. Furthermore, there is a difference between the absolute value of particle size obtained from the XRD analysis and TEM analysis. This discrepancy arise because TEM measures the size of the entire particles, which may consist of several aggregated crystallites, whereas XRD provides the average size of individual coherently diffracting crystallites.^39^

**Fig. 4:**
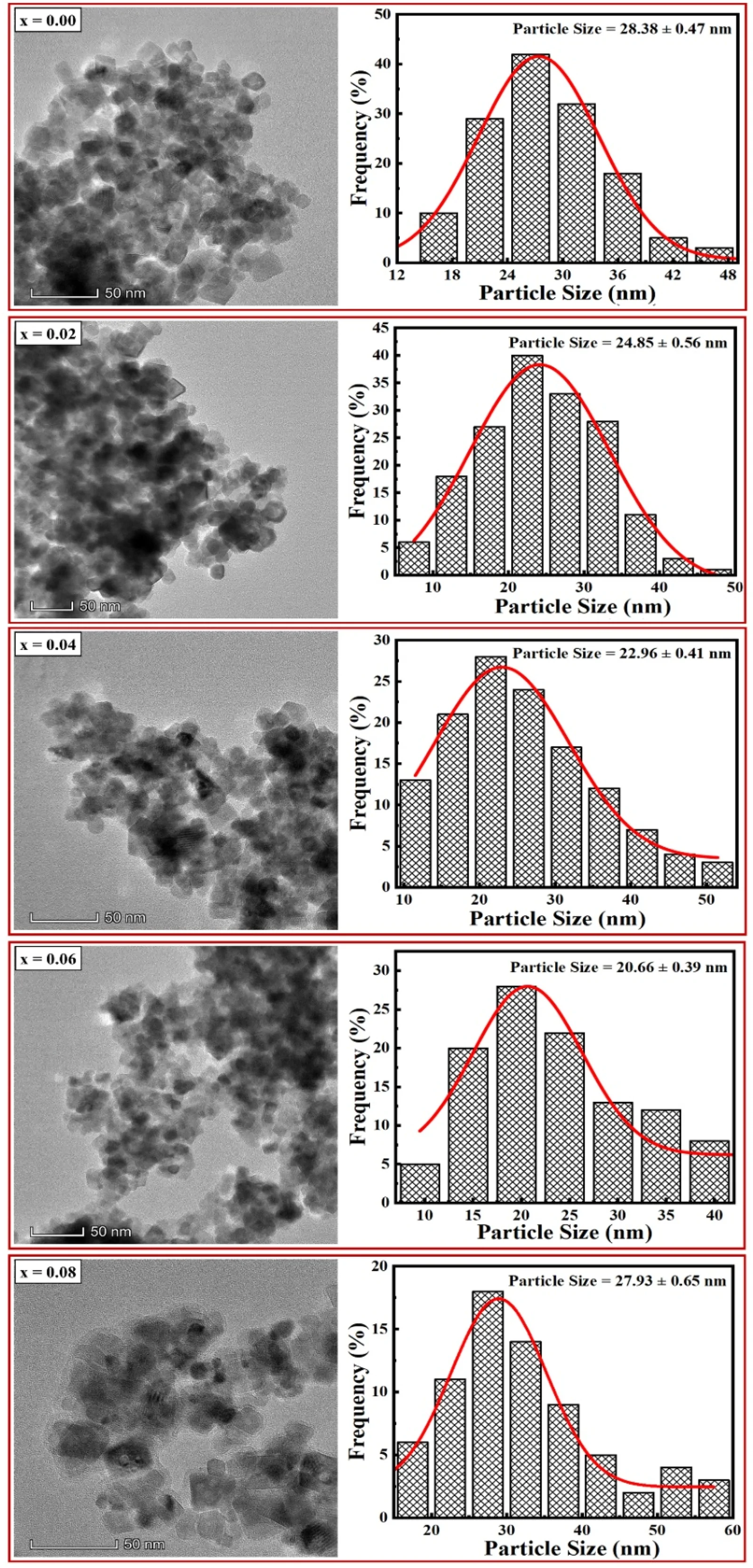
TEM images of Mg_0.4_Zn_0.6_Ho_*x*_Fe_2−*x*_O_4_.

The high-resolution transmission electron microscopy (HRTEM) images were analyzed to investigate the crystallinity and interplanar spacing of the synthesized Mg_0.4_Zn_0.6_Ho_*x*_Fe_2−*x*_O_4_ nanoparticles as represented in Fig. 5. The measured interplanar distances were found to 0.2547, 0.2548, 0.2551, 0.2558 and 0.2542 nm for *x* = 0.00, 0.02, 0.04, 0.06 and 0.08, respectively, which are very close to the interplanar spacing of the (311) crystallographic plane of cubic spinel ferrite, calculated from XRD data.

**Fig. 5:**
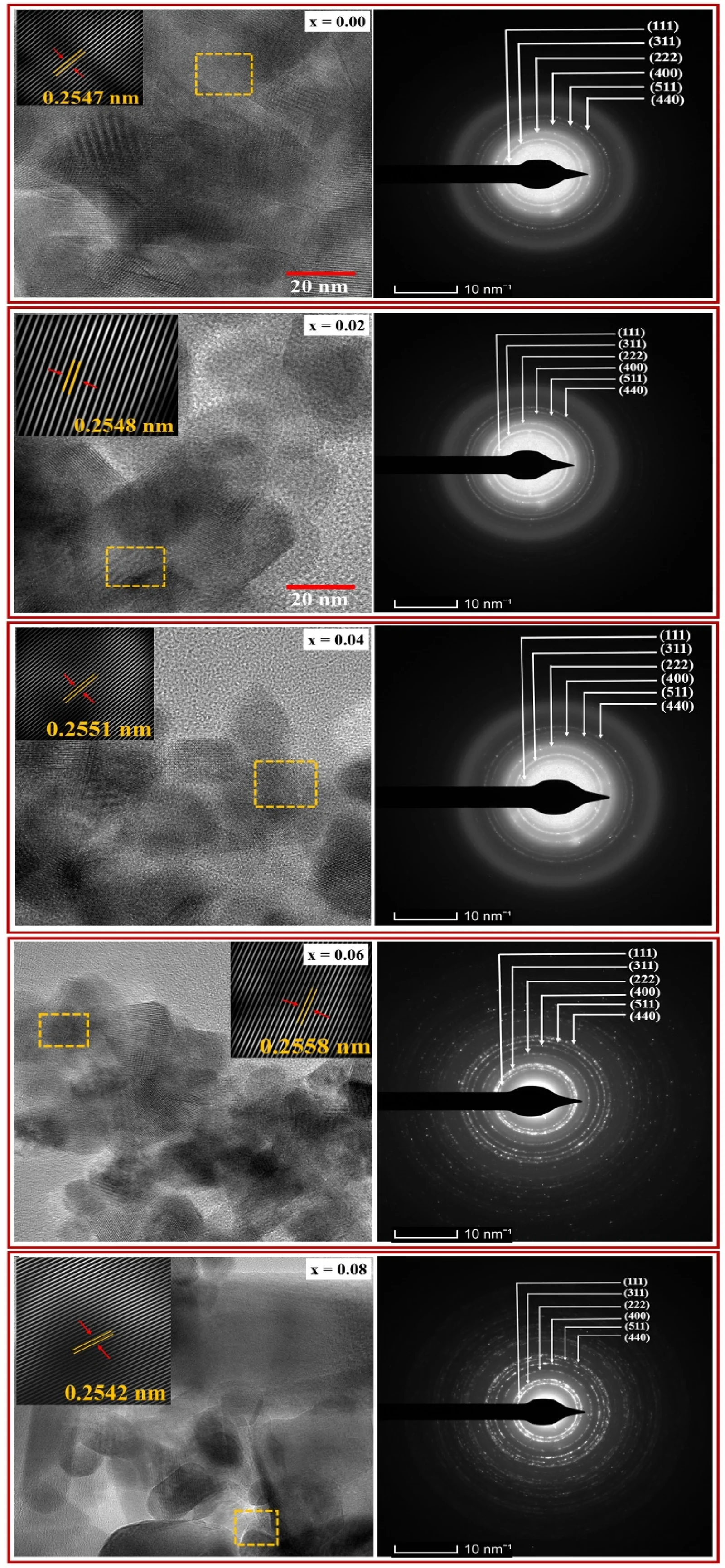
HRTEM and SAED patterns of Mg_0.4_Zn_0.6_Ho_*x*_Fe_2−*x*_O_4_ nanoferrites.

### 3.3 Surface morphology

The micrographs at Fig. 6 reveal that all the samples consist of nearly spherical-shaped nanoparticles with a noticeable degree of agglomeration, which is attributed to the high surface energy of nanosized particles and strong interparticle interactions.^40^ The average grain size was estimated using ImageJ software. The corresponding grain size distribution histograms show that the average grain size gradually increases from 32.64 ± 0.34 nm (*x* = 0.00) to 40.89 ± 0.10 nm for *x* ≤ 0.06. The increase in grain size indicates enhanced grain growth, which may be associated with the densification of the samples resulting in fewer magnetic free pores.^41^ However, a slight reduction in average grain size to 31.64 ± 0.37 nm is observed for *x* = 0.08. This might have happened due to the segregation of excess Ho ions at the grain boundaries after exceeding the solubility limit, which inhibits further grain growth and introduces intragranular porosity, thereby reducing both the density and the average grain size. Energy dispersive spectroscopy (EDS) analysis have been carried out to determine the element composition of the synthesized samples. Fig. 7 shows the elemental distribution mapping and EDS spectra of the undoped (*x* = 0.00) Mg–Zn ferrite and Ho-doped sample (*x* = 0.06). The element compositions of Mg_0.4_Zn_0.6_Ho_*x*_Fe_2−*x*_O_4_ nanoparticles for both compositions are presented in Table 7. The EDS results confirm the presence of Mg, Zn, Fe, O and Ho elements in the studied samples. The exchange of Ho and Fe contents are in consistent in the composition. Meanwhile, the Mg, Zn and O contents remain nearly unchanged, indicating that Ho ions are successfully incorporated into the ferrite lattice. Moreover, no traceable impurity elements were detected, indicating the high purity of the synthesized samples.^42^ The elemental maping images further reveal a uniform distribution of all the elemets throughout the selected areas.

**Fig. 6:**
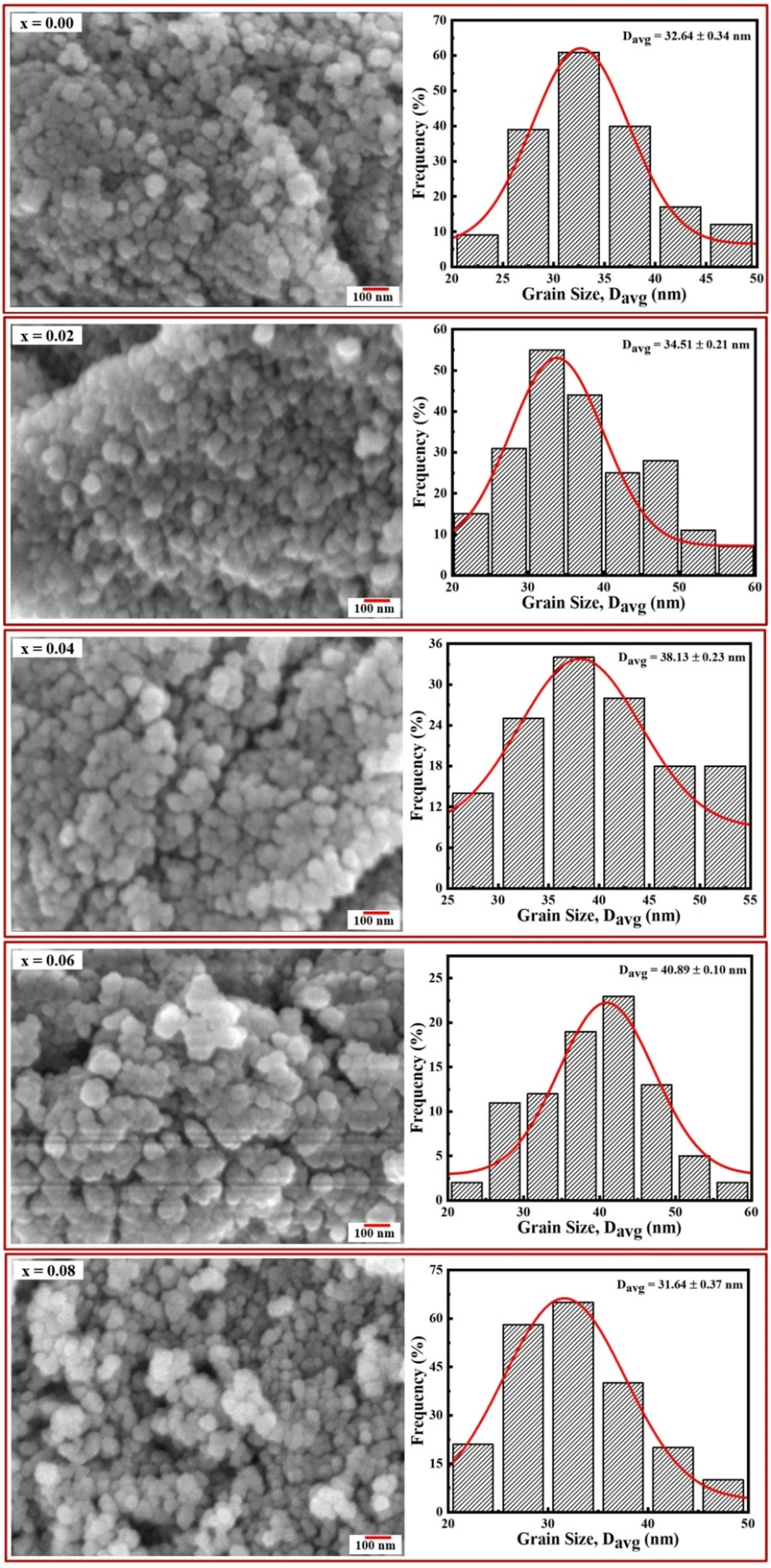
FESEM images and grain size distribution for different Mg_0.4_Zn_0.6_Ho_*x*_Fe_2−*x*_O_4_.

**Fig. 7:**
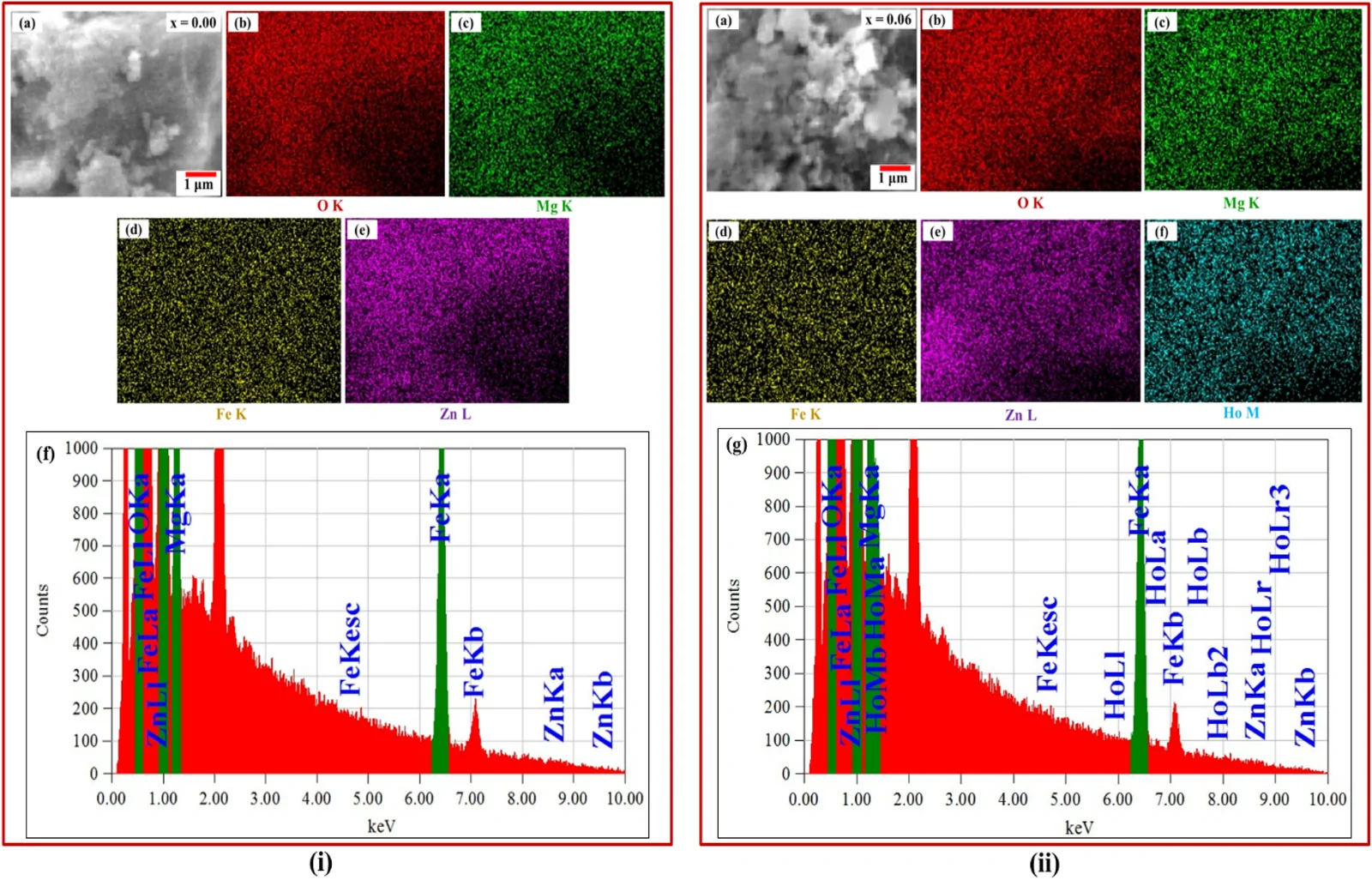
Elemental distribution mapping (O, Mg, Fe, Zn and Ho) and EDS spectra of Mg_0.4_Zn_0.6_Ho_*x*_Fe_2−*x*_O_4_ nanoparticles for (i) *x* = 0.00 and (ii) *x* = 0.06.

**Table 7:** Element composition of Mg_0.4_Zn_0.6_Ho_*x*_Fe_2−*x*_O_4_ nanoparticles for (i) *x* = 0.00 and (ii) *x* = 0.06 in mass%

Composition (*x*)	Mg	Zn	Fe	Ho	O
0.00	4.42	19.38	45.19	0.00	31.00
0.06	4.35	18.86	34.67	10.91	31.51

### 3.4 FTIR analysis

Fourier Transform Infrared (FTIR) sprctroschopy was employed to investigate the formation of the spinel ferrite structure and to identify the characterestistic metal–oxygen (M–O) vibrational modes in Mg_0.4_Zn_0.6_Ho_*x*_Fe_2−*x*_O_4_ (*x* = 0.00–0.08). The FTIR spectra represented in Fig. 8 exhibit two prominent absorption bands, confirming the successful formation of the cubic spinel ferrite structure. The higher-frequency band (*ν*_1_) appears at approximately 532 cm^−1^, is assigned to the stretching vibration of metal–oxygen bonds at the tetrahedral (A) sites, whereas the lower-frequency band (*ν*_2_), appearing in the range 362–369 cm^−1^, corresponds to the stretching vibration of metal–oxygen bonds at the octahedral (B) sites.^43^ The position of the *ν*_1_ band remains almost unchanged for all compositions, indicating that Ho substitution has a negligible influence on the tetrahedral environment. In contrast, the *ν*_2_ band gradually shifts towards lower wavelength from 369 cm^−1^ to 362 cm^−1^ with increasing Ho concentration up to *x* = 0.06, indicating that the introduced Ho ions preferentially occupy the octahedral sites of the spinel ferrite lattice. This red shift is attributed to the substitution of larger Ho ions for Fe ions at the octahedral sites. The larger ionic radius of Ho expands the local crystal lattice, increases the average metal–oxygen bond length and introduces lattice distortion, thereby reducing the bond strength and lowering the vibrational wavenumber.^44^ For the *x* = 0.08 sample, *ν*_2_ shifts slightly towards a higher wavenumber (365 cm^−1^), which may be associated with the appearance of a secondary phase thar partially modifies the local octahedral environment.

**Fig. 8:**
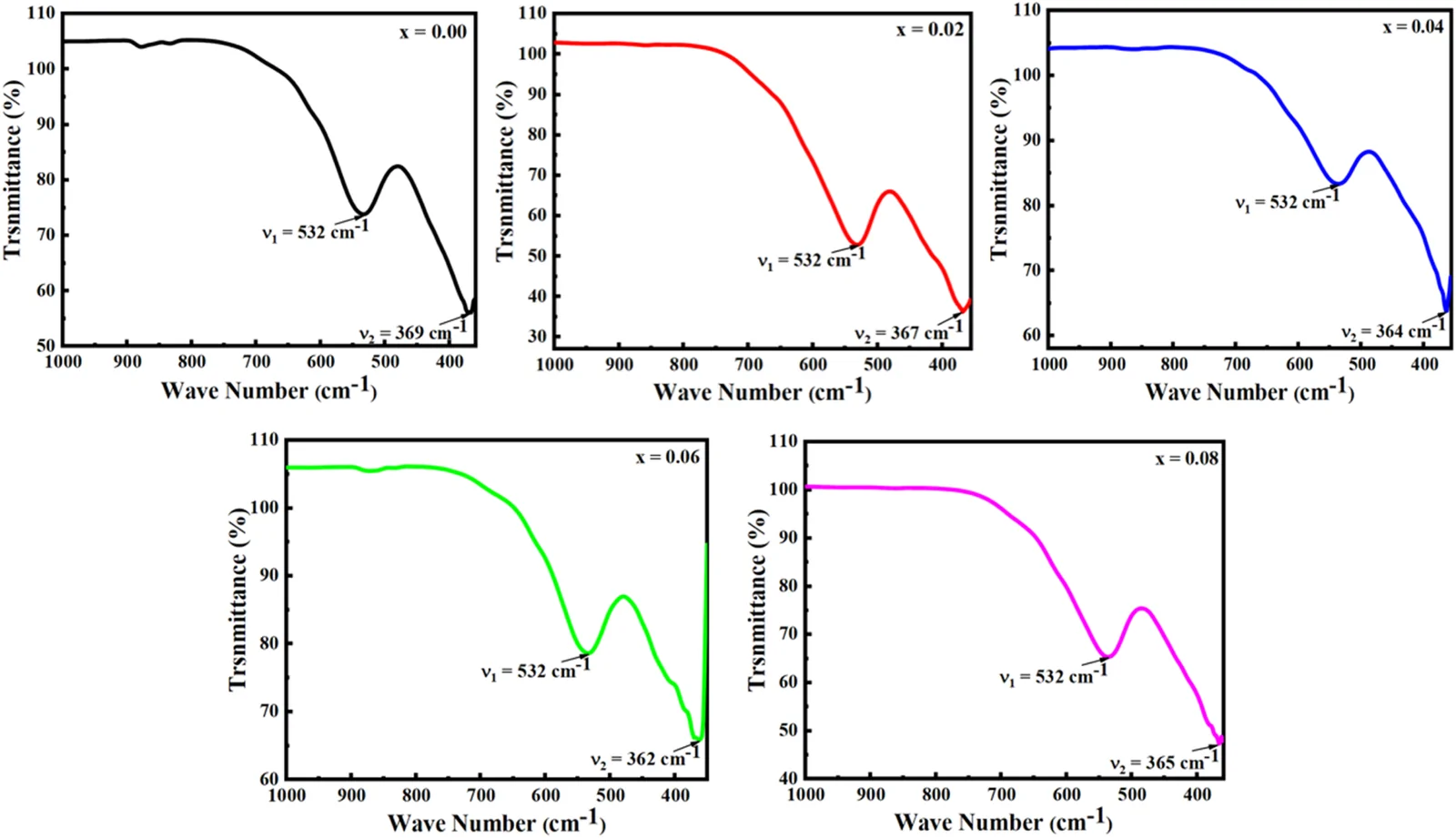
FTIR spectra of Mg_0.4_Zn_0.6_Ho_*x*_Fe_2−*x*_O_4_ (*x* = 0.00, 0.02, 0.04, 0.06 and 0.08) nanoparticles.

The investigation of mechanical properties is essential for these types of functional materials, as it provides valuable insight into their structural stability, durability and suitability for practical applications.^45^ To evaluate the bond strength quantitatively, tetrahedral (*k*_T_) and octahedral (*k*_O_) force constants were calculated using eqn (3.19) and (3.20), while the average force constant *K*_avg_ was determine using eqn (3.21).^46^

3.19 *K*_T_ = 4π^2^*c*^2^*ν*_1_^2^µ

3.20 *K*_O_ = 4π^2^*c*^2^*ν*_2_^2^µ

3.21
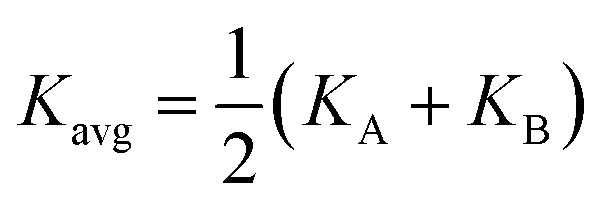
The calculated values are listed in Table 8. It is observed that the tetrahedral force constant (*K*_T_) remains nearly constant, reflecting the invariant tetrahedral vibration observed in FTIR spectra. On the other hand, the octahedral force constant (*K*_O_) decreses gradually from 1.1066 × 10^5^ to 1.0951 × 10^5^ dyne per cm as the Ho concentration increses up to *x* = 0.06. Consequently, the average force constant (*K*_avg_) also decreases slightly. This behavior confirms that Ho incorporation weakens the octahedral metal–oxygen bonds, which is in excellent agreement with the increase in lattice parameter obtained from XRD analysis. The lattice parameter increases from 8.421 (*x* = 0.00) to 8.441 Å (*x* = 0.06), indicating lattice expansion caused by the larger Ho ions. Since longer bond lengths correspond to weaker interatomic interactions, the restoring force acting on the vibrational ions decreases, resulting in lower FTIR vibrational frequencies and reduced force constants.^47^ At *x* = 0.08, both *K*_O_ and *K*_avg_ exhibit a slight increase, which may be attributed to the structural influence of the secondary phase. The weakening of the octahedral metal–oxygen bonds has important implications for the physical properties of the ferrite. A reduction in bond strength weaken Fe–O–Fe superexchange interaction between magnetic ions, thereby influencing the magnetic behavior.

**Table 8:** Two FTIR absorption bands of the ferrites (*ν*_1_) and (*ν*_2_), and force constants (tetrahedral *k*_T_, octahedral *k*_O_, and average *k*_avg_)

Sample (*x*)	*ν*_1_ (cm^−1^)	*ν*_2_ (cm^−1^)	*K*_T_ dyne per cm (10^5^)	*K*_O_ dyne per cm (10^5^)	*K*_avg_ dyne per cm (10^5^)
0.00	532	369	2.1206	1.1066	1.6136
0.02	532	367	2.1206	1.0978	1.6092
0.04	532	364	2.1206	1.0832	1.6019
0.06	532	362	2.1206	1.0743	1.5974
0.08	532	365	2.1206	1.0951	1.6078

### 3.5 Optical properties

#### 3.5.1 Optical band gap energy (*E*_g_)

The absorption spectra of all the synthesized samples are presented in Fig. 9. It is observed that all the samples exhibit relatively strong absorption throughout the investigated spectral region, indicating their ability to interact effectively with incident electromagnetic radiation. The absorption spectra show a broad maximum around 380–450 nm, followed by a gradual decrease in absorbance with increasing wavelength. This behavior is associated with electronic transitions between localized energy states within the crystal lattice.^48^ From Fig. 9 it is clear that the pure sample (*x* = 0.00) exhibits the lowest absorbance, whereas the absorbance gradually increases and reaches its maximum value (∼0.865) for *x* = 0.06. This enhancement in absorbance can be attributed to the incorporation of Ho ions into the spinel lattice, which modifies the electronic structure of the material. The partially filled 4f orbitals of Ho contribute to the formation of localized energy levels near the band edges, thereby increasing the probability of photon absorption.^49^ However, a slight reduction in absorbance is observed for the *x* = 0.08 sample. The formation of a secondary phase indicates a partial solubility limit of Ho ions in the spinel lattice, which can reduce the effective incorporation of Ho into the ferrite structure and consequently diminish the enhancement of optical absorption. The absorption maxima are located at approximately 384.21, 387.08, 400.20, 440.94 and 382.43 nm for *x* = 0.00, 0.02, 0.04, 0.06 and 0.08, respectively. The gradual shift of the absorption maximum toward longer wavelengths up to *x* = 0.06 indicates a red shift, suggesting a reduction in the optical band gap. Howerver, the *x* = 0.08 sample shows a slight blue shift, which may be attributed to the formation of the secondary Ho_2_O_3_ phase.

**Fig. 9:**
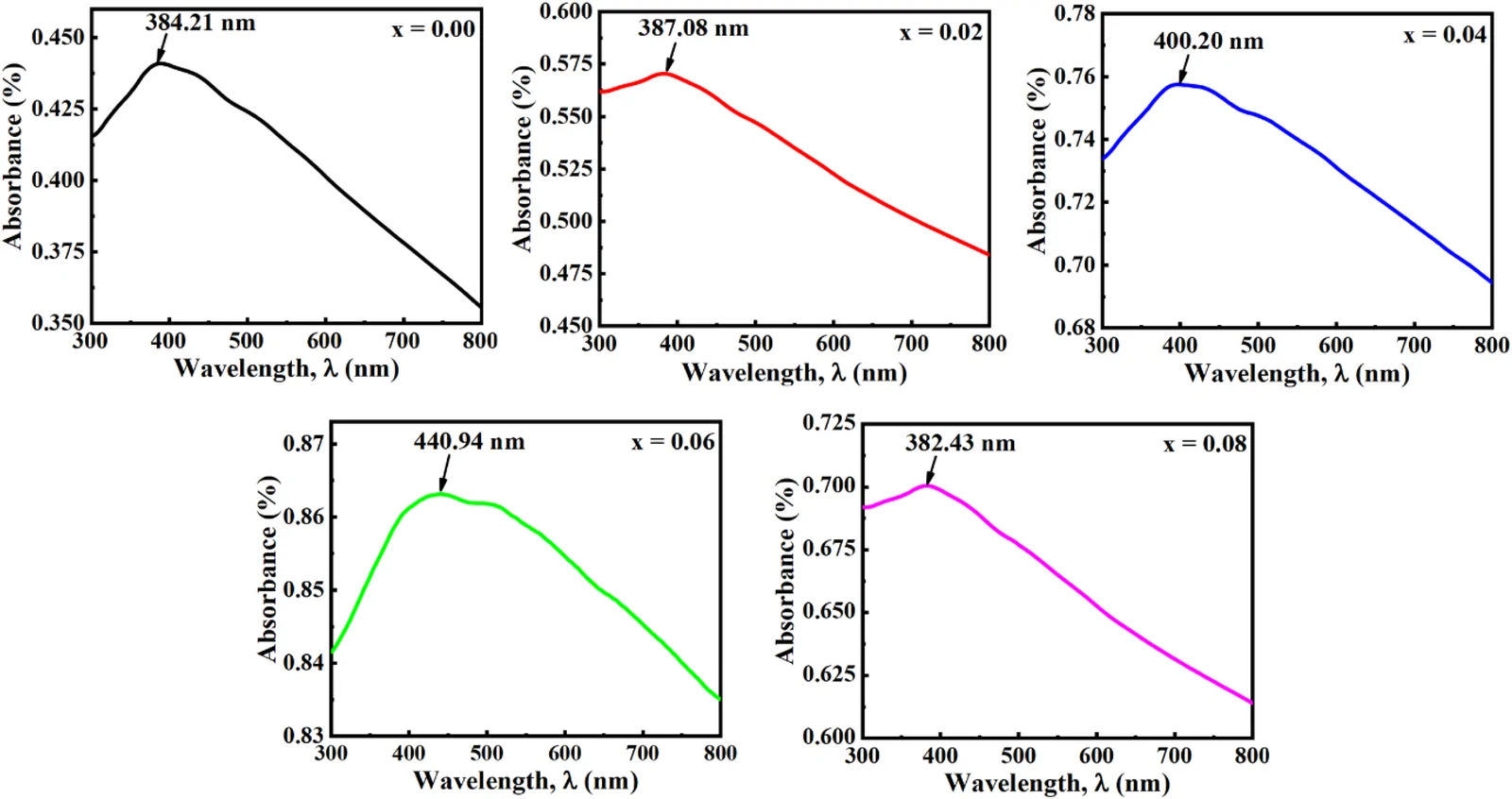
UV-visible spectrum.

The optical band gap energy (*E*_g_) of the synthesized samples was determined using the Tauc relation:^50^

3.22 (*αhν*)^*n*^ = *A*(*hν* − *E*_g_)

where *α* is the absorption coefficient, *hν* is the photon energy, *A* is a material-dependent constant and *E*_g_ represents the optical band gap energy. When *n* = 2, it corresponds to allowed indirect transitions. The band gap values were estimated by extrapolating the linear region of the (*αhν*)^2^*versus hν* plots to the energy axis, as shown in Fig. 10 and the obtained band gap values are summarized in Table 9. The result reveals that the value of band gap decreases from 1.62 for the pristine ferrite to 1.45 eV for *x* = 0.06 composition. This relatively larger band gap of the undoped sample may be associated with the intrinsic electronic structure of ferrite, where the interaction between Fe-3d and O-2p orbitals determines the energy separation between the valence and conduction bands. The gradual reduction of the band gap energy with Ho substitution up to *x* = 0.06 can be explained by the modification of the electronic structure caused by the presence of Ho-related energy states. The partially filled 4f states of Ho ions can introduce additional localized levels within the forbidden energy region, which act as intermediate states for electronic excitation. Furthermore, the substitution of larger Ho ions induces lattice distortion and defect formation, producing localized states near the band edges. These effects decrease the energy required for electron transition, resulting in the observed narrowing the band gap.^51^ However, at higher Ho concentration (*x* = 0.08), a slight increase in the band gap is observed. This can be due to the formation of a secondary phase at higher Ho concentration. The increase in Ho concentration may enhance the carrier concentration, giving rise to the Burstein–Moss effect. In this phenomenon, the lower-energy states near the bottom of the conduction band become occupied by excess charge carriers. As a result, electrons from the valence band can no longer be excited into these filled states in the conduction band. Consequently, the optical absorption edge shifts toward higher energies (blue shift), leading to an apparent increase in the measured optical band gap.^52^ The comparative band gap values of synthesized Mg_0.4_Zn_0.6_Ho_*x*_Fe_2−*x*_O_4_(*x* = 0.00, 0.02, 0.04, 0.06 and 0.08) samples with different conventional semiconductors are presented in Table 9. The obtained band gap energy in the range 1.45–1.65 eV, confirms the narrow band gap semiconductor nature of the present ferrite system. Notably, the *x* = 0.06 sample exhibits the lowest band gap energy (1.47 eV), which is very close to the reported values of typical narrow band gap semiconductors such as GaAs (∼1.42 eV) and AlGaAs (∼1.45 eV). This comparable band gap facilitates efficient visible-light absorption and enhances photo-induced electron–hole pair generation. Therefore, the optimized Mg_0.4_Zn_0.6_Ho_0.06_Fe_1.54_O_4_ sample can be considered a promising visible-light-responsive semiconductor for photocatalytic, photoelectrochemical, solar energy conversion and optoelectronic applications.

**Fig. 10:**
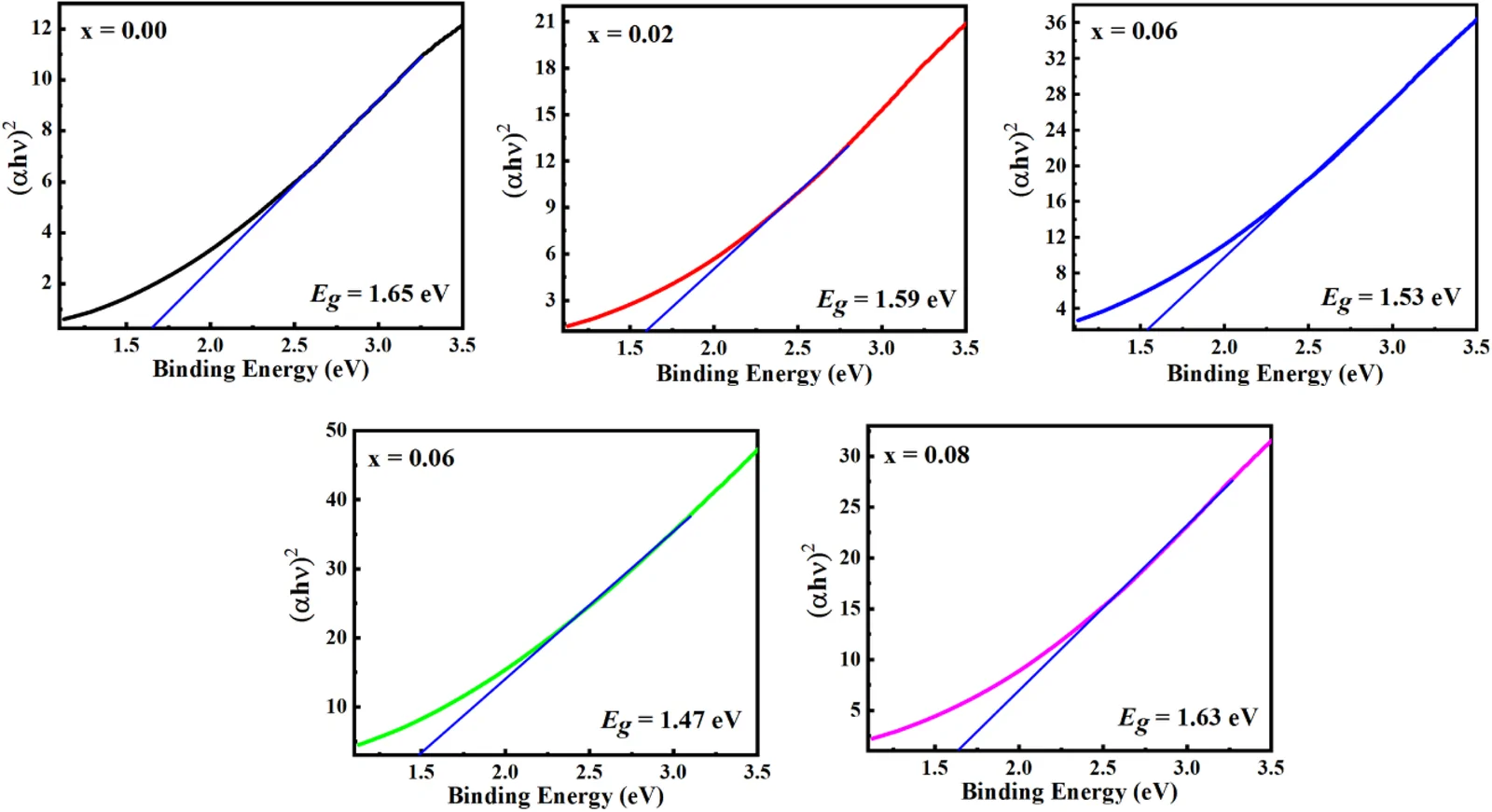
(*αhν*)2 *vs.* photon energy.

**Table 9:** Comparison of optical band gap energy of Mg_0.4_Zn_0.6_Ho_*x*_Fe_2−*x*_O_4_ ferrite with different semiconductor material

Sample	Band gap energy (eV)	Semiconductor type	References
Ge	0.66	Narrow band gap semiconductor	53
Si	1.12	Narrow band gap semiconductor	54
GaAs	1.42	Narrow band gap semiconductor	55
AlGaAs	1.45	Narrow band gap semiconductor	56
Mg_0.4_Zn_0.6_Fe_2_O_4_	1.65	Narrow band gap semiconductor	This work
Mg_0.4_Zn_0.6_Ho_0.02_Fe_1.98_O_4_	1.59	Narrow band gap semiconductor	This work
Mg_0.4_Zn_0.6_Ho_0.04_Fe_1.96_O_4_	1.53	Narrow band gap semiconductor	This work
Mg_0.4_Zn_0.6_Ho_0.06_Fe_1.94_O_4_	1.47	Narrow band gap semiconductor	This work
Mg_0.4_Zn_0.6_Ho_0.08_Fe_1.92_O_4_	1.63	Narrow band gap semiconductor	This work
CdSe	1.74	Narrow band gap semiconductor	57
CdS	2.42	Moderate band gap semiconductor	58
ZnO	3.37	Wide band gap semiconductor	59

### 3.6 Magnetic properties

#### 3.6.1 Saturation magnetization and coercivity

The magnetic hysteresis (M–H curve) of the studied samples at room temperature is represented in Fig. 11. The saturation magnetization (*M*_s_), remanent magnetization (*M*_r_), coercivity (*H*_c_) and anisotropy constant (*K*_a_) have been calculated and summarized in Table 10. The hysteresis loops confirmed the *S*-shaped ferrimagnetic nature of all synthesized samples. It is observed that the value of *M* decreases from 25.5 (for *x* = 0.00) to 21.7 emu g^−1^ (for *x* = 0.06). The law of approach has been used to find the value of saturation magnetization (Ms) for all the sampes. It is tabulated in Table 10. According to the Goodenough–Kanamori–Anderson (GKA) theory, the strength of exchange interaction depends strongly on the bond length and bond angles. As the Fe–O bond lengths increase, the overlap between Fe (3d) and O (2p) orbitals decreases, resulting in the reduction of the exchange integral for magnetic coupling.^60^ Simultaneously, the observed variation of bond angles indicates a gradual distortion of the Fe–O–Fe exchange geometry. These structural modifications weaken the A–B superexchange interaction,^61^ resulting in a lowering the saturation magnetization. Moreover, the larger Ho ions introduce lattice distortion which promotes spin canting in the B-sublattice, reducing the net magnetization.^62^ A similar trend is also observed for *M*_r_ and the value of *M*_r_ decreases from 0.77 to 0.53 emu g^−1^ for *x* ≤ 0.06. This can be attributed to the weakening of A–B superexchange interaction and increased spin canting, which reduce the stability of magnetic domain alignment after removal of the applied magnetic field, thereby lowering the remanent magnetization.^63^ The magnetocrystalline anisotropy constant (*K*_a_) has been calculated using the following relation and presented in Table 10.3.24
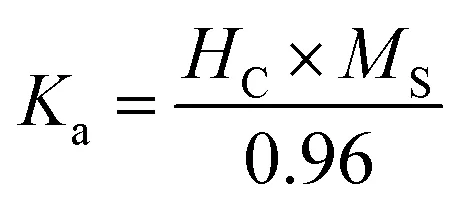


**Fig. 11:**
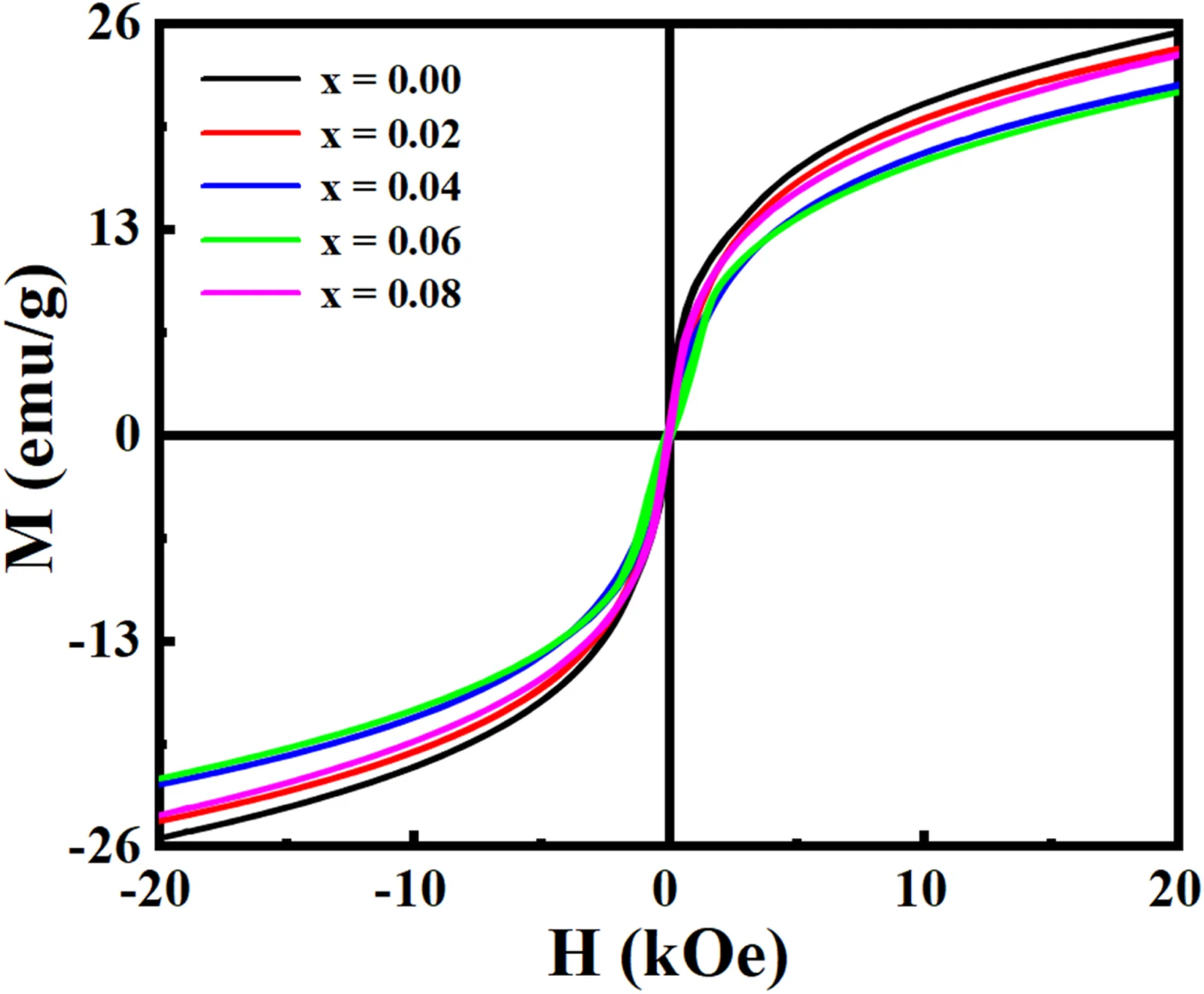
Variation of *M* with the applied magnetic field strength, *H*.

**Table 10:** Magnetization (*M*) at ±20 kOe, saturation magnetization (*M*_s_), retentivity (*M*_r_), coercivity (*H*_c_) and anisotropy constant (*K*_a_) for different compositions

Sample (*x*)	*M* (emu g^−1^)	*M*_s_ (emu g^−1^)	*M*_r_ (emu g^−1^)	*H*_c_ (Oe)	*K*_a_ (erg cm^−3^)
0.00	25.5	26.17	0.77	33.9	897
0.02	24.3	25.59	0.69	65.7	1664
0.04	22.1	23.80	0.61	96.7	2221
0.06	21.7	23.62	0.53	134.4	3032
0.08	23.9	25.09	0.62	67.9	1694

It is clear that the value of *K*_a_ increases from 897 (for *x* = 0.00) to 3032 erg cm^−3^ (for *x* = 0.06), followed by a noticeable decrease to 1694 erg cm^−3^ at *x* = 0.08. Moreover, the larger ionic radius of Ho induces lattice distortion and internal strain, which further enhances anisotropy through magnetoelastic effect.^64^ Furthermore, at *x* = 0.08, the value of *K*_a_ decreases which can be due to the solubility limit of Ho ions in spinel structure. At higher doping level, cation redistribution, local clustering of Ho ions and partial relaxation of lattice strain may occur. These effects reduce the effectiveness of spin–orbit interaction and weaken the structural contribution to anisotropy, leading to a reduction in *K*_a_. The coercivity (*H*_c_) has been calculated from M − H curve and it is given in Table 10. The *H*_c_ increases significantly from 33.9 to 134.4 Oe with increasing Ho content up to *x* = 0.06. This enhancement is closely associated with the increase in magnetic anisotropy, which follows the Brown's relation,^65^
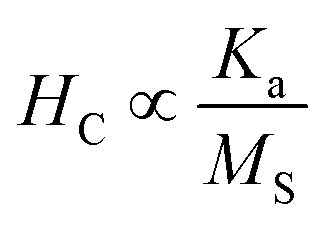
. The variation of saturation magnetization (*M*_s_), coercivity (*H*_c_), and magnetocrystalline anisotropy constant (*K*_a_) with Ho content is shown Fig. 12. It is evident that *M*_s_ decreases with increasing Ho content for *x* = 0.06. In contrast, both *H*_c_ and *K*_a_ increase with Ho content, attain a maximum at *x* = 0.06. The Ho ion exhibits a strong preference at the octahedral (B) site, which modifies the cation distribution and the strength of the Fe^3+^ (A)–O^2−^–Fe^3+^ (B) super-exchange interaction. Furthermore, because the 4f electrons of Ho are highly localized and shielded by the outer 5s and 5p shells, the orbital overlap between Ho 4f and O 2p orbitals is weak, resulting in a feeble Ho^3+^–O^2−^–Fe^3+^ super exchange interaction. The progressive weakening of the A–B exchange interaction with increasing Ho content gives rise to a non-collinear spin arrangement in the B-sublattice. On the other hand, the simultaneous increase in *H*_c_ and *K*_a_ with Ho substitution up to *x* = 0.06 can be attributed to the progressive reduction in particle size with increasing Ho content which inhibits crystal growth. The Ho substitution introduce strong spin–orbit coupling and lattice strain, which enhances the magnetocrystalline anisotropy of the system. This increased anisotropy raises the energy barrier for domain wall motion, thereby increasing coercivity.^66^ However, at *x* = 0.08, both anisotropy constant and coercivity show a decreasing trend. This behavior can be attributed to the formation of a secondary Ho_2_O_3_ phase. The segregation of Ho ions reduces their contribution to the host lattice anisotropy and weakens the effective spin–orbit coupling. As a result, both magnetocrystalline anisotropy and domain wall pinning diminished, leading to lower *K*_a_ and *H*_c_ values.

**Fig. 12:**
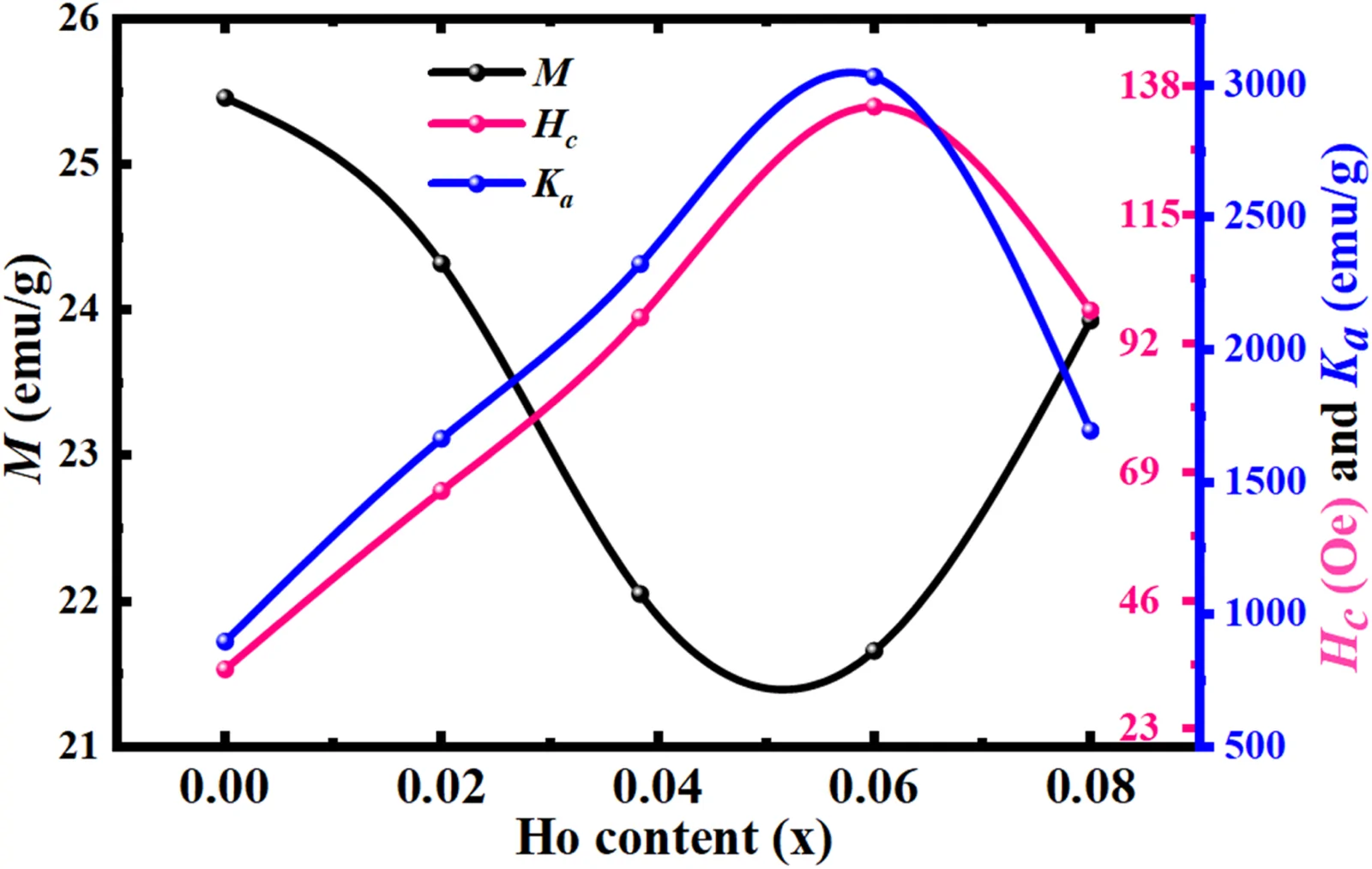
Variation of *M*, *H*_c_ and *K*_a_ with Ho content.

### 3.7 AC electrical transport properties

#### 3.7.1 Dielectric constant and loss tangent

The frequency-dependent dielectric constant (*ε*′) of Mg_0.4_Zn_0.6_Ho_*x*_Fe_2−*x*_O_4_ (*x* = 0.00, 0.02, 0.04, 0.06 and 0.08) measured at 300 K is shown in Fig. 13. For all the investigate compositions, (*ε*′) decreases significantly with increasing frequency and gradually approaches a nearly constant value at higher frequencies. This behaviour can be explained based on the Maxwell–Wagner interfacial polarization mechanism.^67^ According to this model, ferrites can be considered as electrically heterogeneous materials consisting of well conducting grains separated by poorly conducting grain boundaries. As the frequency increses, the applied electric field changes more rapidly and the charge carriers cannot follow the field effectively. Therefore, the contribution of interfacial polarization decreses, resulting in a reduction of *ε*′. The frequency dependence of the dielectric constant can also be described by the Debye relation:^68^3.25
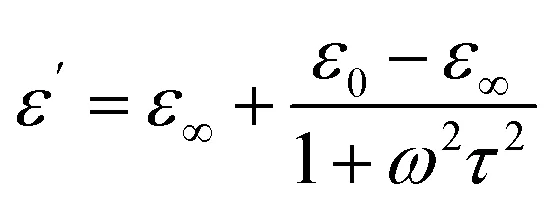
where *τ* is the relaxation period, *ε*_0_ represents the dielectric constant at extremely low frequency, *ε*_∞_ stands for the dielectric constant at high frequency. The observed decrease in *ε*′ with frequency is consistent with the polarization mechanism at higher frequencies. It is also seen that *ε*′ increases with increasing Ho content for *x* ≤ 0.06. The could be attributed to the densification of the samples resulting in facilitate interfacial polarization, thereby contributing to the increase in *ε*′. While for *x* = 0.08, the incorporation of excess Ho introduces greater structural distortion by forming a secondary phase and increase the resistive contribution of grain boundaries, which can suppress charge accumulation and consequently reduce *ε*′. In spinel ferrites, electron hopping predominantly occurs at the octahedral-B sites through the Fe^2+^ ↔ Fe^3+^ electronic exchange mechanism. The incorporation of Ho^3+^ ions at B-sites limit the electronic exchange. Consequently, at *x* = 0.08, the reduced Fe^2+^/Fe^3+^ hopping probability suppresses charge-carrier polarization, leading to a lower dielectric constant.

**Fig. 13:**
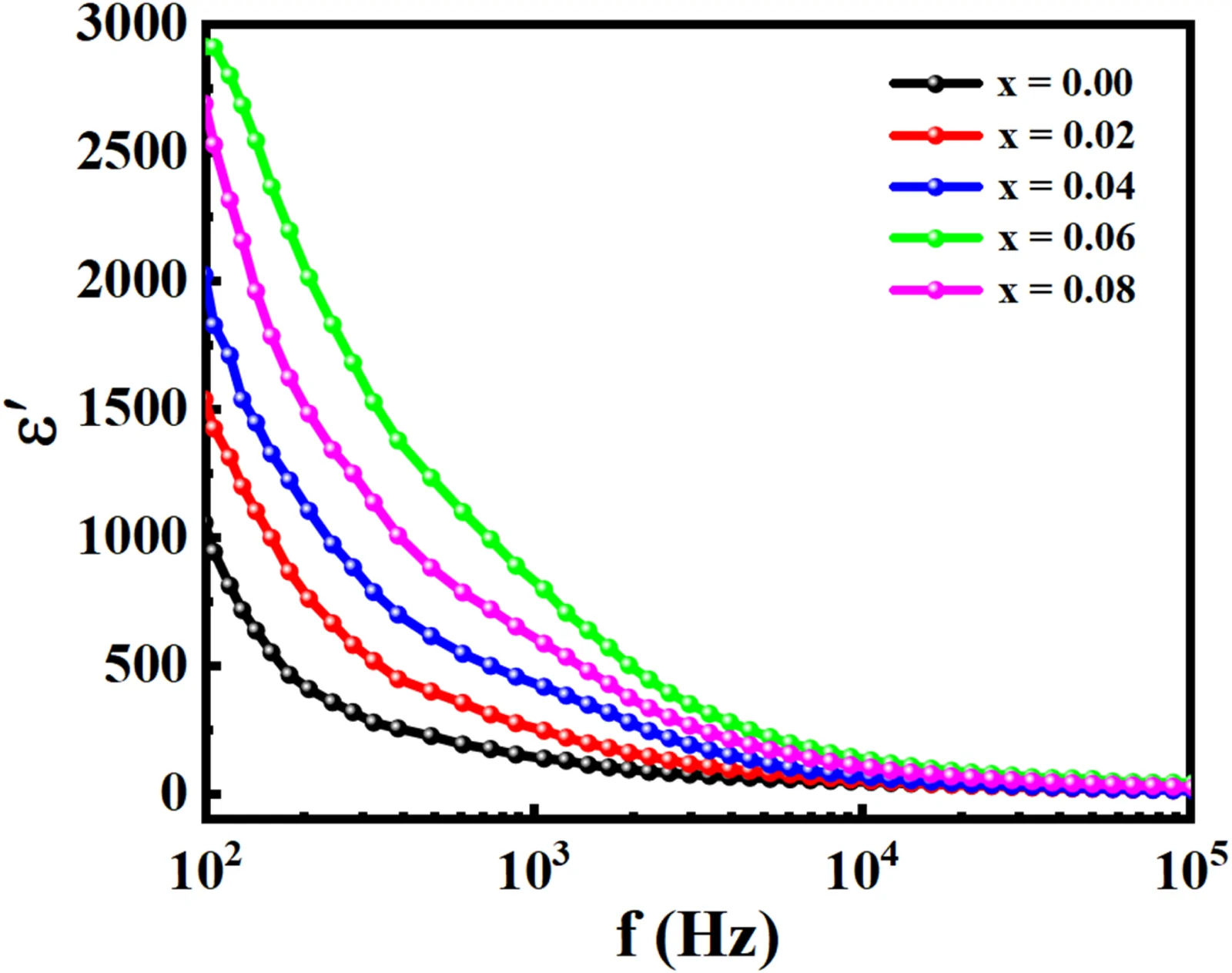
Variation of *ε*′ with an applied frequency.

Fig. 14 illustrates the loss tangent (tan *δ*) of the synthesized samples. The value of tan *δ* gradually decreases with increasing frequency, which may be related to the inability of domain-wall motion with the applied frequency. Ferrimagnetic resonance takes place when the electron hopping frequency becomes comparable to the frequency of the applied field.^69^ In the present study, the resonance peak of the Ho-doped Mg–Zn ferrite with *x* = 0.06 shifts toward a higher frequency compared with the undoped sample. The inclusion of Ho in Mg–Zn ferrites shows the lower value of dielectric losses, which could make them suitable in high-frequency operations.

**Fig. 14:**
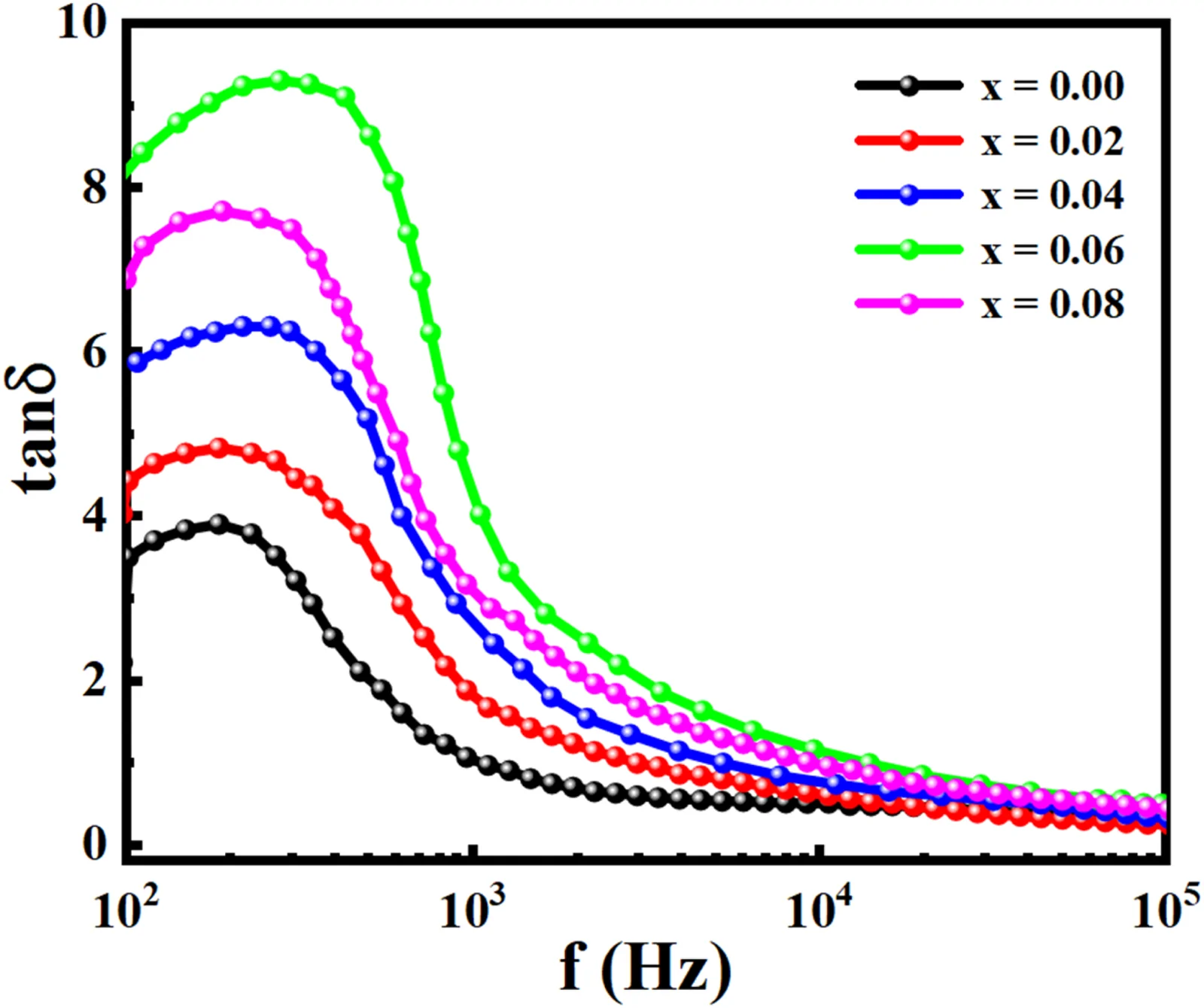
Variation of tan *δ* with an applied frequency.

#### 3.7.2 AC electrical conductivity

Fig. 15 illustrates the frequency dependence of AC electrical conductivity (*σ*_ac_) of the synthesized samples measured at 300 K. At lower frequencies, the conductivity remains relatively low, whereas a pronounced increase is observed with increasing frequency. This frequency-dependent behaviour is associated with the enhanced hopping and mobility of localized charge carriers under the applied alternating electric field. The frequency variation of *σ*_ac_ can be described using Jonscher's universal power law:^70^*σ*_ac_ = *σ*_dc_ + *A*(*T*)*ω*^*n*^, where *σ*_dc_ represents the frequency-independent DC conductivity, *A* is the pre-exponential factor, *ω* is the angular frequency, and *n* is the dimensionless frequency exponent. Among the investigated compositions, the sample with *x* = 0.06 exhibits the larger electrical conductivity. This enhanced conductivity can be attributed to its densification, which reduces the pores and facilitates charge-carrier movement through the ferrite matrix. The larger grain size also promotes the interfacial polarization and charge transport, resulting in a higher AC conductivity. Therefore, the observed variation in AC conductivity is consistent with the corresponding dielectric behaviour of the studied samples.

**Fig. 15:**
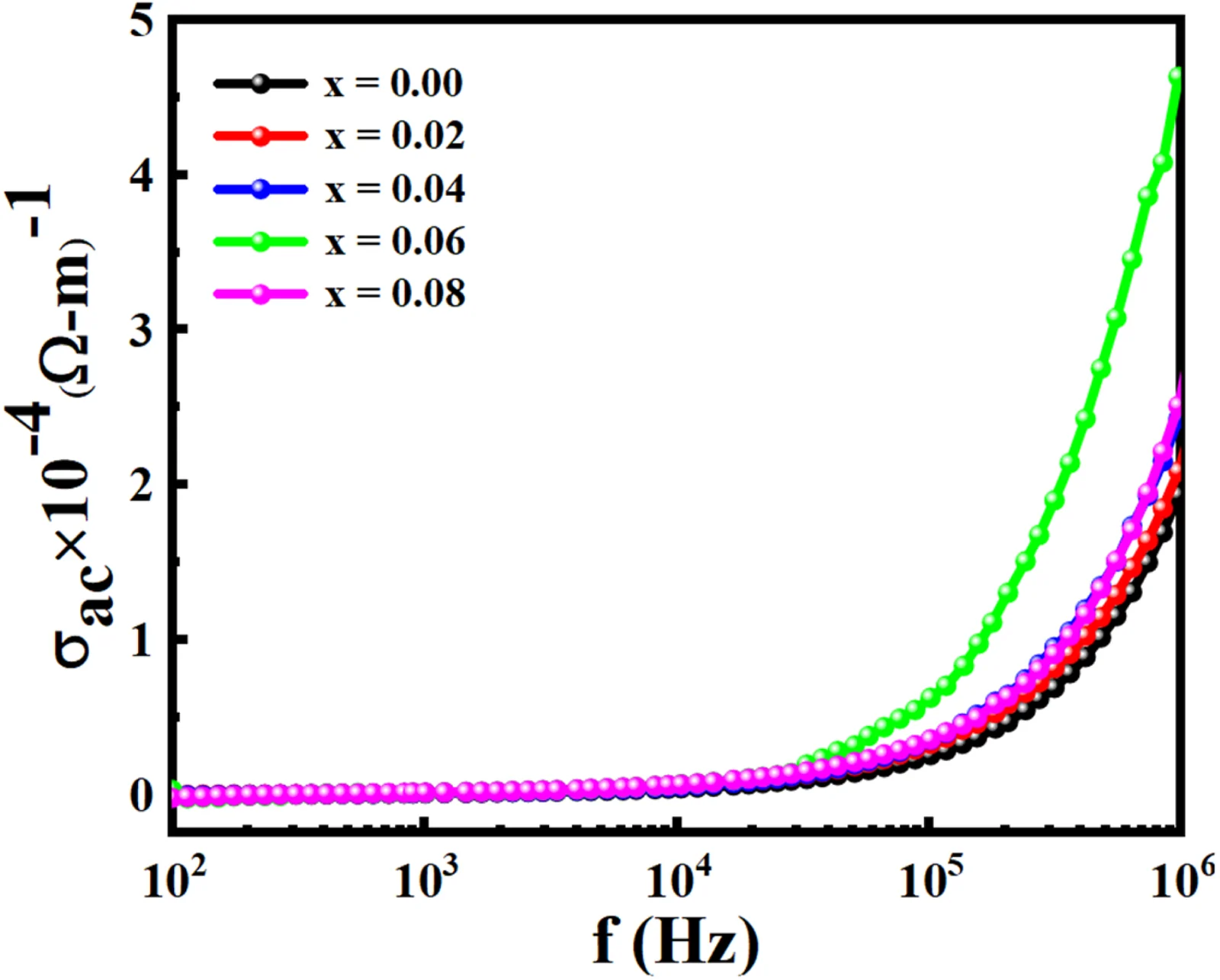
Variation of *σ*_ac_ with the applied frequency.

#### 3.7.3 Cole–Cole plot

The Cole–Cole or Nyquist plot, represented by the relationship between the imaginary (*Z*″) and real (*Z*′) parts of impedance, provides useful information about the electrical contributions of grains, grain boundaries, and electrode interfaces.^71^ For an ideal Debye relaxation process, a single semicircular arc with its centre located on the *Z*′ axis is generally expected. The Cole–Cole plots of the investigated samples are presented in Fig. 16. Compared with the undoped Mg–Zn ferrite, the Ho-substituted samples exhibit a noticeable reduction in the semicircular arc. Among the studied compositions, the sample with *x* = 0.06 displays the smallest arc, indicating lower resistivity and consequently enhanced the electrical conductivity.

**Fig. 16:**
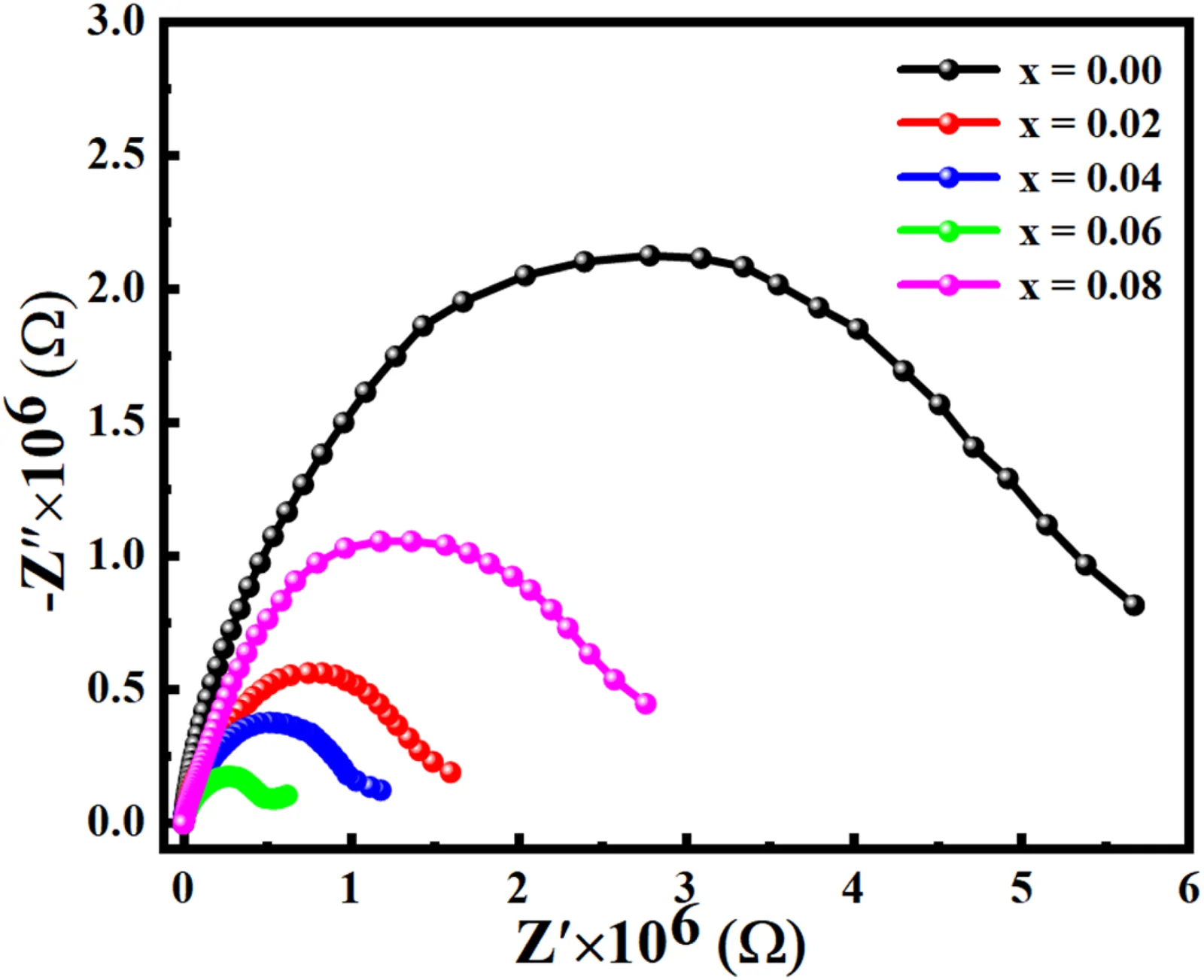
Cole–Cole plot.

#### 3.7.4 Electric modulus

The dielectric relaxation behavior of Ho-doped Mg–Zn ferrite samples was further investigated through complex electric modulus analysis. The complex electric modulus is defined as the reciprocal of the complex dielectric permittivity and can be expressed as:^72^M* = *M*′ + *iM*″
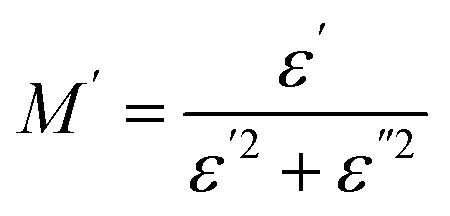

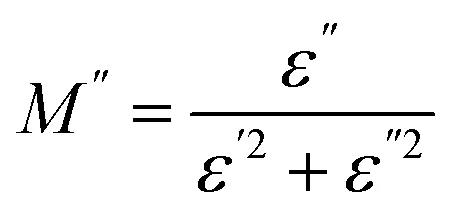
where *M*′ and *M''* are the represent the real and imaginary parts of the electric modulus, respectively. The frequency-dependent variation of *M′* for the Ho-doped Mg–Zn ferrite samples presented in Fig. 17 shows very low values in the low-frequency region and gradually increases with increasing frequency. The low values of *M′* at lower frequencies can be attributed to the dominance of electrode and interfacial polarization arising from the accumulation of charge carriers. At higher frequencies, the charge carriers are unable to follow the rapidly alternating electric field, resulting in a reduction in polarization and consequently increase the modulus.

**Fig. 17:**
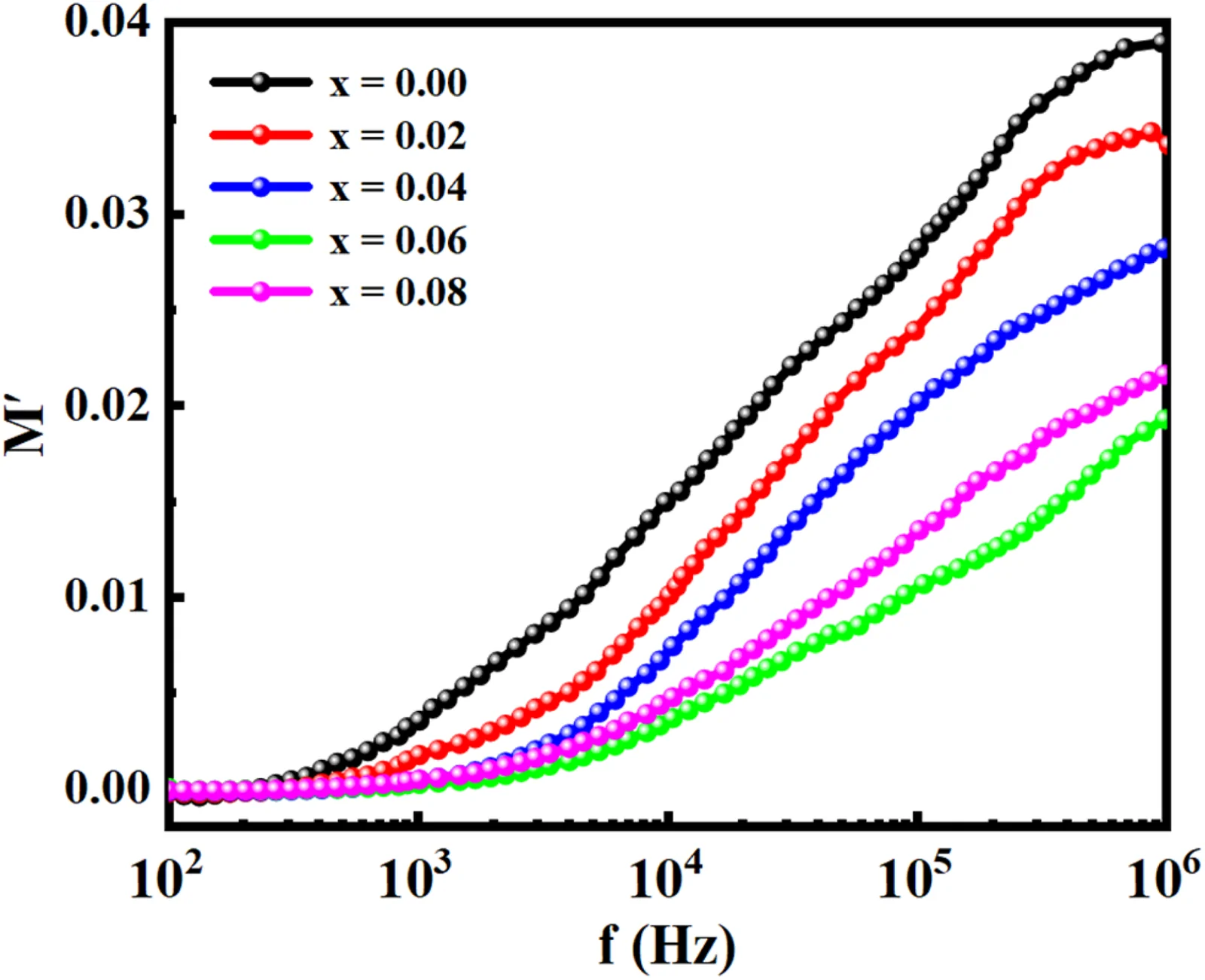
Variation of *M*′ with frequency.

The frequency dependence of *M''* in Fig. 18 provides further insight into the dielectric relaxation mechanism of Ho-doped Mg–Zn ferrite samples. A distinct relaxation peak in the spectrum indicates the presence of a characteristic relaxation process within the material. Such relaxation can be associated with the hopping and localized motion of charge carriers, particularly the electron exchange between Fe^2+^ and Fe^3+^ ions at the octahedral sites. A shift of the (*M''*) peak toward higher frequencies indicates a decrease in relaxation time and hence a faster response of the charge carriers to the applied electric field. Thus, the electric modulus analysis confirms the presence of localized charge-carrier relaxation in the studied samples.

**Fig. 18:**
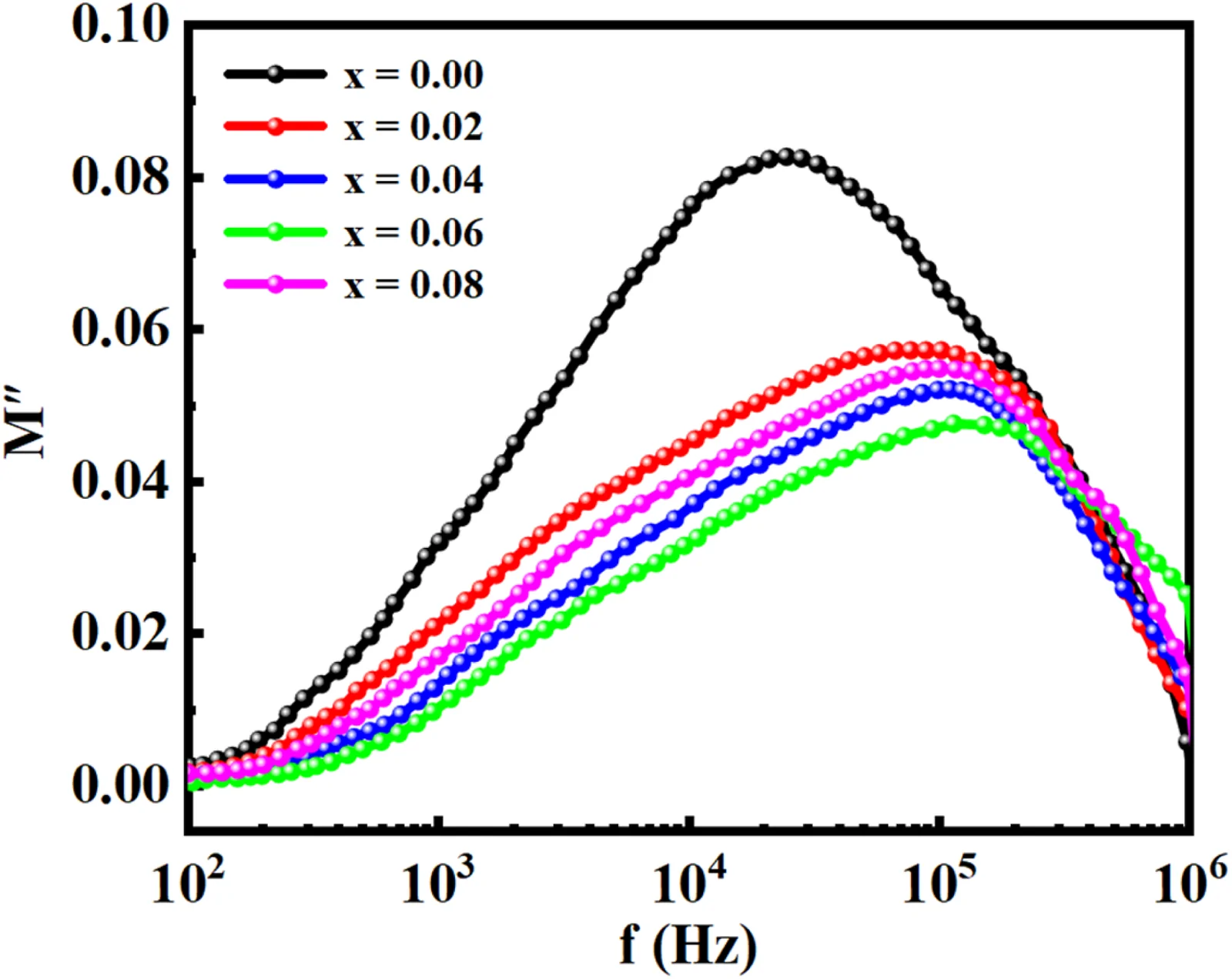
Variation of *M*″ with frequency.

## 4 Conclusions

The present investigation demonstrates that controlled Ho incorporation can significantly modify the multifunctional properties of Mg–Zn ferrite nanoparticles. The structural analysis confirmed the formation of a cubic spinel phase, with the lattice parameter increasing and the crystallite size decreasing with Ho addition up to *x* = 0.06. A weak Ho_2_O_3_ secondary phase was detected at *x* = 0.08, indicating that excessive Ho incorporation is unfavorable for maintaining the single spinel phase. The reduction in crystallite size was also supported by TEM analysis, which showed nearly spherical nanoparticles with particle size decreasing up to *x* = 0.06. The FTIR results confirmed the characteristic metal–oxygen bonds of the spinel structure and showed noticeable changes in the octahedral vibration with Ho substitution. The optical study revealed a decrease in the band gap from 1.65 to 1.47 eV up to *x* = 0.06, suggesting that Ho incorporation can improve visible-light absorption and modify the electronic structure of the ferrite. The magnetic response was strongly influenced by Ho substitution. Although the saturation magnetization decreased from 25.46 to 21.66 emu g^−1^, the coercivity increased from 33.85 to 134.39 Oe, while the magnetic anisotropy constant increased from 897.73 to 3032.17 erg cm^−3^ up to *x* = 0.06. This enhancement indicates increased magnetic anisotropy and improved resistance to domain-wall motion. Consequently, the *x* = 0.06 composition shows greater potential for magnetic recording and data-storage applications where enhanced magnetic stability is desirable. The electrical measurements further demonstrated the beneficial effect of Ho substitution up to *x* = 0.06. The dielectric constant increased with Ho content up to this concentration, while the AC conductivity reached its maximum value. The impedance spectra showed the smallest semicircular arc for *x* = 0.06, indicating lower impedance and improved charge transport. In addition, the electric modulus analysis confirmed frequency-dependent relaxation, with the relaxation peak shifting toward higher frequencies. These characteristics indicate the suitability of the material for high-frequency electronic and electromagnetic applications. Therefore, controlled Ho substitution, particularly at *x* = 0.06, offers a promising route for developing Mg–Zn ferrite nanoparticles for magnetic storage, high-frequency electronic, electromagnetic and optoelectronic applications.

## Author contributions

Muhammad Samir Ullah: conceptualization, data curation, formal analysis, investigation, methodology, writing – original draft & editing and supervision. Kaniz Fatema Laizo: data curation, formal analysis, investigation, writing – review & editing.

## Conflicts of interest

The authors declare that they have no conflict of interest.

## Data Availability

The authors declare that the data supporting the findings of this study are available from the first and corresponding authors on request.
